# Electrochemical CO_2_ Capture by a Quinone-Based Covalent Organic Framework

**DOI:** 10.1021/jacs.5c12304

**Published:** 2025-12-09

**Authors:** Muhammad Abdullah Khan, Zhen Xu, Muhammad Muzammil, Samuel Bird, Monica Munawar, Fariah Salam, Niamh A. Hartley, Jack Taylor, Kamran Amin, Jianheng Ling, Henry R. N. B. Enninful, Naveed Zafar Ali, Kai Hetze, Sijia Cao, Yan Lu, Zhixiang Wei, Martin Oschatz, Phillip J. Milner, Alexander C. Forse

**Affiliations:** † Yusuf Hamied Department of Chemistry, 2152 University of Cambridge, Lensfield Road, Cambridge CB2 1EW, U.K.; ‡ Renewable Energy Advancement Laboratory, Department of Environmental Sciences, 66757 Quaid-i-Azam University, Islamabad 45320, Pakistan; § CAS Key Laboratory of Nanosystems and Hierarchical Fabrication, National Center for Nanoscience and Technology, Chinese Academy of Sciences, Beijing 100190, P. R. China; ∥ Department of Chemistry and Chemical Biology, Cornell University, Ithaca, New York 14850, United States; ⊥ Felix Bloch Institute for Solid State Physics, Faculty of Physics and Earth Sciences, Leipzig University, Linnéstraße, 504103 Leipzig, Germany; # National Centre for Physics, Quaid-i-Azam University, Islamabad 44000, Pakistan; ∇ Institute for Technical Chemistry and Environmental Chemistry, 9378 Friedrich-Schiller-University Jena, Philosophenweg 7a, 07743 Jena, Germany; ○ Helmholtz Institute for Polymers in Energy Applications Jena, Lessingstraße 12-14, 07743 Jena, Germany; ◆ 28340 Institute of Electro chemical Energy Storage, Helmholtz-Zentrum Berlin für Materialien und Energie, Hahn-Meitner-Platz 1, 14109 Berlin, Germany; ¶ Department of Materials and Henry Royce Institute, University of Manchester, Manchester M13 9PL, U.K.

## Abstract

Electrochemical CO_2_ capture is an emerging technology that promises to be more energy-efficient than traditional thermal or pressure-swing processes. Herein, the first evidence of electrochemical capture of CO_2_ using a covalent organic framework (COF) is presented. We hypothesized that the assembly of anthraquinone units into a well-defined porous framework electrode would lead to enhanced electrochemical CO_2_ capture compared to previous approaches that grafted anthraquinones on carbon supports and suffered from low CO_2_ capacities and stabilities. To test this, an anthraquinone-based COF is employed, and it is found that the quinones are electrochemically accessible for reversible CO_2_ capture in an ionic liquid electrolyte. The system achieves a high electrochemical CO_2_ uptake capacity >2.6 mmol g^–1^ COF, reaching half of the theoretical CO_2_ capacity of the material and surpassing the capacities of anthraquinone-functionalized carbons. The stability and CO_2_ uptake rate issues encountered with the ionic liquid system are also addressed by using aqueous electrolytes where we attained stable carbon capture for 500 cycles with a 99.6% Coulombic efficiency and an electrical energy consumption of 31 kJ mol_CO_2__^–1^. The use of covalent organic framework electrodes can become a general strategy for understanding and enhancing the electrochemical CO_2_ capture.

## Graphical abstract

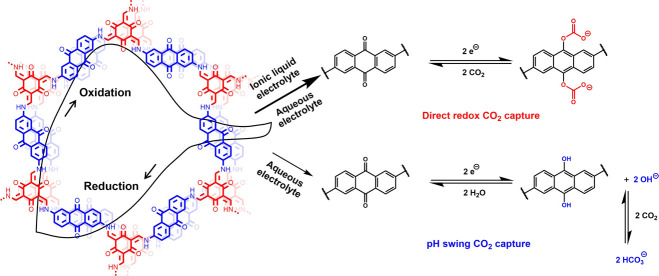

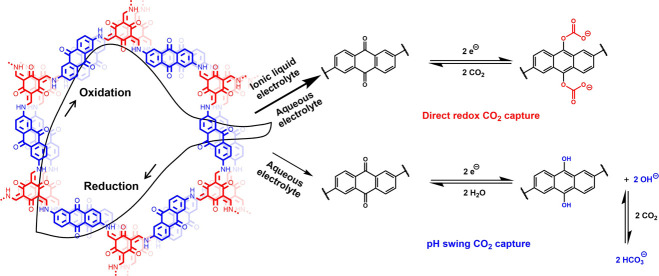

## Introduction

Energy-efficient CO_2_ capture is vital for urgent climate change mitigation. As a promising alternative to traditional CO_2_ capture methods, electrochemical CO_2_ capture (eCC) employing switchable redox-active carriers is gaining significant momentum.^1–5^ The research in this area is progressing in several directions including (i) the development of stable capture systems with higher CO_2_ uptake capacities and (ii) the discovery of new solid sorbents that can operate at high current densities and are compatible with aqueous electrolytes.^1,6–8^ On the first strand, by leveraging the redox behavior of active molecular disulfides, bipyridyls, thiolates, and extensively studied quinones, a diverse range of capture systems have been developed.^9^ Even the simplest representatives of the quinone family have an appealingly high theoretical capacity of two CO_2_ equivalents per molecule, compared to conventional amines which require 2 equiv of amine to capture 1 equiv of CO_2_.^1,2,6^ Importantly, eCC offers the benefit of conducting CO_2_ capture and release without the need for external heating or heat removal and has thus shown promising energy efficiencies.^2,10–12^

Efforts to integrate solid CO_2_ sorbents into eCC have seen remarkable initial successes. A notable example is the immobilization of polyanthraquinones on carbon nanotubes (CNTs) to build a semisolid faradaic swing system that demonstrated an impressive cyclic performance in both ionic liquid (IL) and water-in-salt electrolytes.^13,14^ Building on this work, we successfully grafted anthraquinone (AQ) units onto conductive carbon substrates, achieving a 50% charge utilization of the loaded redox moieties for CO_2_ capture. However, this system suffered from rapid CO_2_ capacity loss over time, and it was challenging to control and characterize the quinone loading.^15^ Furthermore, existing redox-based eCC systems often face challenges related to structural integrity, slow CO_2_ uptake kinetics, and low mass loadings, imposing optimization and operational constraints.^2,4,16,17^

Motivated by the progress presented above, we hypothesized that the CO_2_ affinity seen in molecular quinones could be replicated in quinone-based covalent organic framework (COF) materials. The electrochemical reduction of these materials would generate phenoxide anions, which could then bind electrophilic CO_2_, while the subsequent electrochemical oxidation would liberate CO_2_ and regenerate the quinone (Figure 1). We propose that COF-based redox systems have a high density of well-defined active capture sites (the anthraquinone units), while the high COF porosity would enable the required rapid CO_2_ and electrolyte transport. Additionally, the diversity of constituent redox moieties and COF structures offer possibilities to fine-tune the CO_2_ uptake performance, while the use of earth-abundant C, N, H, and O elements is beneficial from a sustainability standpoint.^18–20^ We note that one very recent study also explored a COF for electrochemical carbon dioxide capture, but ultimately did not observe any electrochemical capture by the phenazine framework which was explored, while it was also unclear to what extent the phenazine units were redox-active in the studied electrochemical cell.^21^ The authors instead proposed the use of phenazine macrocycles as the redox-active capture materials, with these materials demonstrating accessible redox reactions, as well as electrochemically driven CO_2_ capture and release.^21^ Importantly, their study highlighted the key challenge of achieving good electron transport in covalent-framework-type materials, which we have addressed further below.

**Figure 1:**
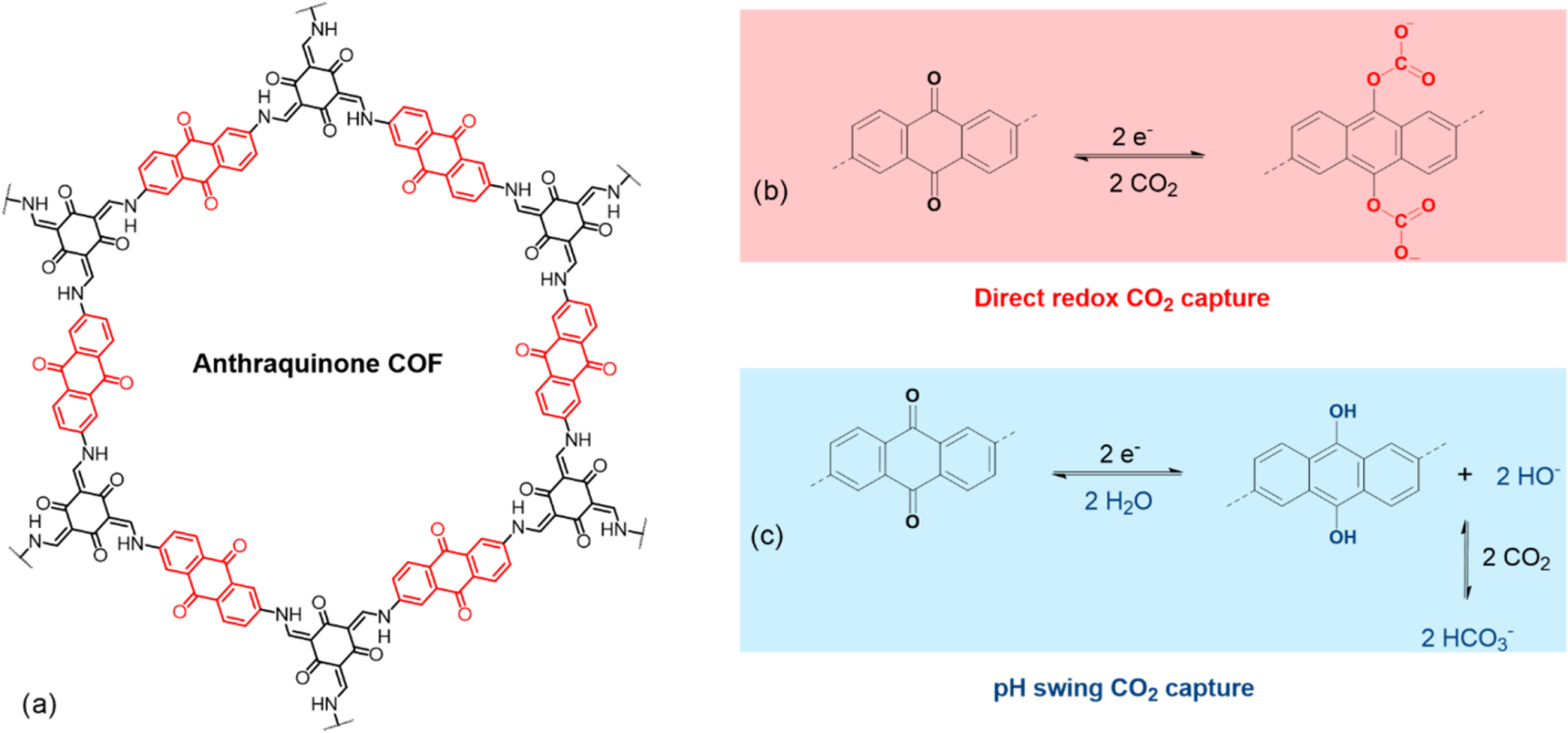
Proposed mechanisms of electrochemical CO_2_ capture by an anthraquinone-based covalent organic framework. (a) Structure of the anthraquinone COF studied in this work. (b) “Direct redox” capture mechanism and (c) “pH swing” capture mechanism.

## Results and Discussion

To test the hypothesis in Figure 1, we synthesized a well-known anthraquinone-based COF (AQCOF) and employed it in electrochemical CO_2_ capture experiments.^22^ Details on the synthesis, characterization of the AQCOF, electrode preparation, and device assembly can be found in the Supporting Information (Sections S1–S4 and Figures S1–S6). In brief, our two-electrode battery-like cells are equipped with an AQCOF–carbon composite as the working electrode, an activated carbon counter electrode to balance the charge, 1-butyl-3-methylimidazolium bis­(trifluoromethylsulfonyl)­imide ionic liquid ([Bmim]­[TFSI], IL) as the electrolyte, and a pressure sensor to monitor electrochemical sorption and any irreversible pressure change associated with side reactions within the CO_2_ environment of the cell.

When the cells were charged to negative voltages (i.e., by inducing the reduction of the AQCOF material), a CO_2_ pressure drop was observed, supporting the electrochemical capture of CO_2_ by the anthraquinone units (Figure 2a). A subsequent discharge back to 0 V led to a pressure increase ascribed to the electrochemical oxidation of the quinone units and CO_2_ release. Importantly, the changes in the CO_2_ pressure closely followed the applied cell voltage over repeated cycles, demonstrating that the cycle is reversible (Figure S7). From the observed pressure change, an initial uptake capacity of ∼1.5 mmol g^–1^ CO_2_ uptake per gram of the AQCOF was calculated.

**Figure 2:**
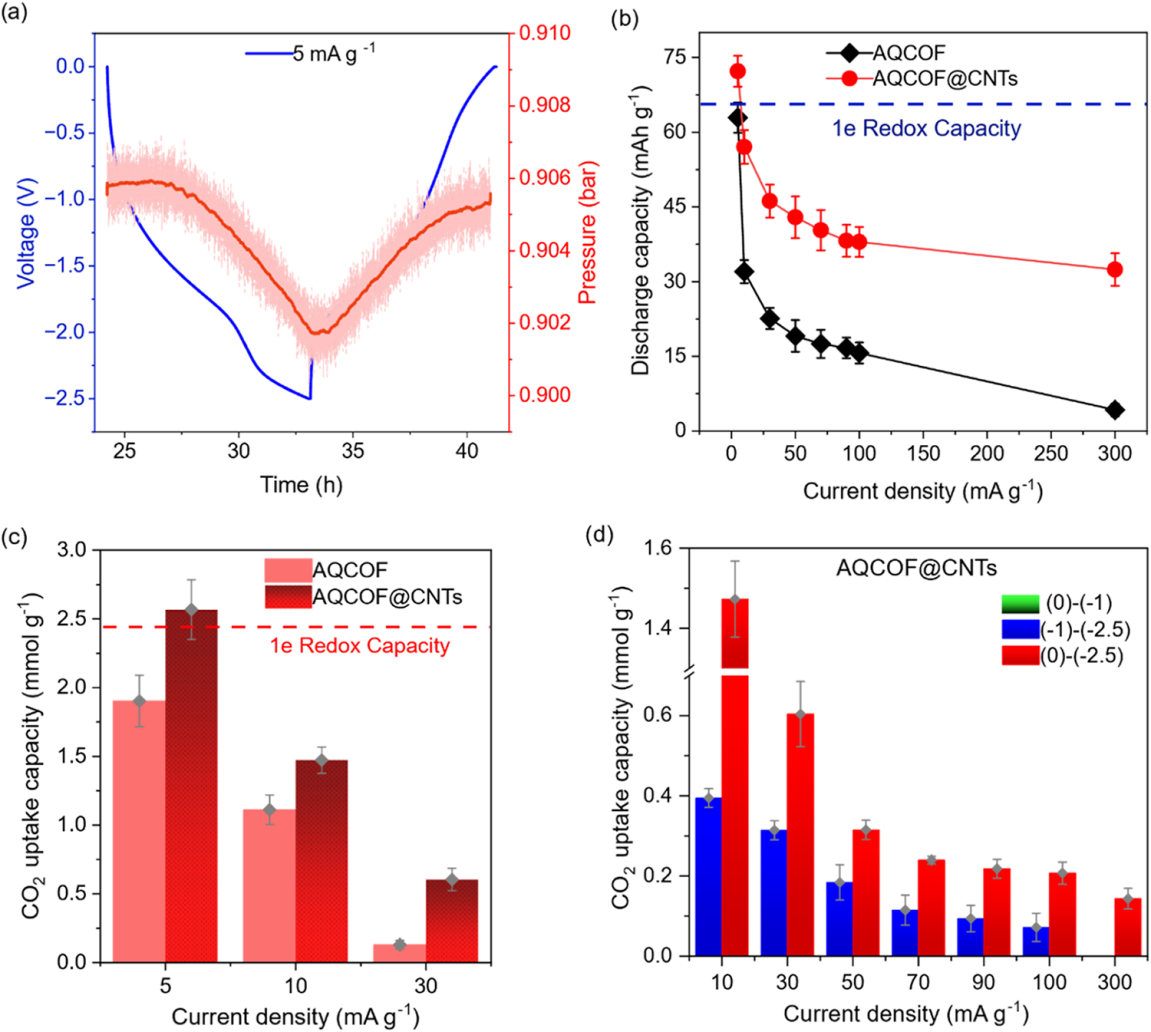
Electrochemical CO_2_ capture performance of AQCOF-based capture systems using [Bmim]­[TFSI] as the electrolyte. (a) A typical electrochemical galvanostatic discharge curve showing CO_2_ pressure changes (smoothed pressure curve, moving average every 100 s, red line) at a 5 mA g^–1^ constant current in static mode and with a 5 min voltage hold. (b) Electrochemical discharge capacities under CO_2_ at different current densities and (c) CO_2_ uptake capacities at low current densities, with data shown for electrodes made from the pristine AQCOF and electrodes made from AQCOF@CNT materials. (d) Effect of breaking the working voltage window into different voltage regimes on electrochemical CO_2_ uptake capacity using AQCOF@CNTs.

An improvement in the conductivity and charge capacity of the material was achieved by growing the AQCOF on carbon nanotubes (CNTs).^23^ This addresses the limited accessibility of redox-active sites that often plagues COF–carbon composites.^21^ To implement this in the device, the composition with 7% CNTs following AQCOF synthesis (denoted hereafter as AQCOF@CNTs) with a specific surface area (SSA) of 325 m^2^ g^–1^ was selected for its minimal surface area difference with the pristine AQCOF (SSA 310 m^2^ g^–1^). Electrodes fabricated with the AQCOF–carbon nanotube composite exhibited improved charge storage capacities compared to the AQCOF alone (Figure 2b) and attained 1.09 e^–^ (74 mAh g^–1^ at 5 mA g^–1^) and ∼0.85 e^–^ (58 mAh g^–1^ at 10 mA g^–1^) charge capacities per anthraquinone unit, indicating that quinone units remote from the conductive carbon additives might be electrically isolated and therefore not accessed.

Further insights into the electrochemical CO_2_ uptake performance were obtained by varying the current density during charge–discharge cycles (Figure 2c). In general, a high CO_2_ uptake is observed only at low current densities. Notably, at a current density of 5 mA g^–1^, the CO_2_ uptake reached ∼2.6 mmol g^–1^ for AQCOF@CNTs. This CO_2_ uptake capacity is the best among all known capacitive or redox electrochemical capture systems where direct comparison is possible.^8,15,21,24,25^ Fast charging shows a decreasing trend in both the CO_2_ uptake and the charge capacity, while right-shifted pressure curves portray a delayed CO_2_ uptake in response to the applied cell voltage (Figure S8a–c). This observation further supports our hypothesis of direct redox binding. The ideal electron-to-CO_2_ ratio for system study is 1 (each anthraquinone unit can undergo two electron reductions, which can in principle capture two CO_2_ molecules). We found that the number of CO_2_ molecules captured per electron stored decreases progressively and the electron/CO_2_ utilization ratio at 5, 10, and 30 mA g^–1^ for CO_2_ capture falls from 0.87 and 0.64 to 0.34 e^–^, respectively (Figure S9), inferring that at higher current densities, the CO_2_ reaction in the COF channels and ion movement can no longer sustain the reaction rate and can limit the CO_2_ uptake in the cell.

The evidence of redox CO_2_ capture by the anthraquinone units was gathered with measurements in different voltage windows, along with cyclic voltammetry (CV) scans from two-electrode cells (Figure S10a,b). The CV of the AQCOF@CNTs under CO_2_ exhibits two distinct features (Figure S10b). Within the voltage range 0 to −1 V, a narrow rectangular capacitive feature devoid of redox activity appears. Afterward, as the voltage was increased from −1 to −2.25 V, a broad peak became evident, assigned to anthraquinone reduction. This information combined with the galvanostatic charge–discharge profile presented in Figure 2a was then used to assess how voltage windows affect the CO_2_ uptake performance. As shown in Figure 2d, when the cell was operated within the purely capacitive range of 0 to −1 V, no pressure change occurred (Figure S11a), suggesting that quinone redox is essential for driving electrochemical CO_2_ capture by the AQCOF. Instead, when limiting the voltage between −1 and −2.5 V, i.e., in a range of prominent redox activity, the CO_2_ pressure oscillated periodically in response to the applied voltage (Figure S11b). Nonetheless, the uptake remained lower than what was recorded across the full 0 to −2.5 V range. Low CO_2_ uptake at higher charging rates also indicated the need for more oxidizing potentials and more time to fully regenerate quinones to drive the next capture cycle. Furthermore, applying positive polarization from 0 to 2.5 V (i.e., positively charging the COF electrode) raised the pressure inside the cell with abrupt pressure changes, suggesting the degradation of the material (Figure S12). Subsequent operation of the cell within 0 to −2.5 V showed no pressure change, indicating that the cell was no longer functional. Our previous experiments with the electrochemical cell employing porous carbon electrodes as both electrodes and the same ionic liquid electrolyte exhibited only a very minor electrochemical CO_2_ uptake,^15^ supporting the idea that the CO_2_ capture process by the AQCOF is predominantly driven by anthraquinone redox.

To assess our system under more realistic conditions, we evaluated the uptake capabilities in different gas mixtures. For 100% CO_2_ and a 15% CO_2_ mixture with N_2_, CO_2_ uptake capacities were within error of each other (Figure S13a–d). To investigate whether the pressure changes were indeed due to CO_2_ capture, measurements were conducted under 100% N_2_ and showed only very small pressure changes (Figure S14). While small periodic pressure changes were observed under 100% N_2_, they remained the same in response to different current densities, and quantified values fell within the measurement uncertainty. We attribute these small changes to the movement of electrolyte ions within the COF channels and possible electrolyte density changes due to electrochemical charging. The O_2_ sensitivity of anthraquinones is well documented.^6,26,27^ Experiments under O_2_-containing atmospheres (15% O_2_/20% CO_2_/60% N_2_; Figure S15a–d) show significant performance deterioration. We assumed the pressure changes come solely from CO_2_ uptake (release), rather than N_2_ uptake (release); CO_2_ uptake decreased to 0.09 ± 0.02 mmol g^–1^ at 100 mA g^–1^ and 0.08 ± 0.03 mmol g^–1^ at 50 mA g^–1^, respectively. Compared with Coulombic efficiencies under 100% CO_2_ (97.6% at 100 mA g^–1^ and 95.3% at 50 mA g^–1^), Coulombic efficiencies under oxygen at the same current densities dropped to 91.2 and 86.3%, respectively. The lower uptake at lower charging rates and the decreases in Coulombic efficiencies suggest that redox-active sites are reoxidized by oxygen, making them unavailable for CO_2_ uptake, consistent with the known oxygen sensitivity of anthraquinone systems.^6,26,27^ The observed O_2_ sensitivity highlights an important design challenge for redox-active framework systems, and future optimization should extend studies to full flue-gas compositions, containing SO_*x*_ and NO_*x*_. Finally, for stability evaluation, in long cycling experiments, the system was charged with an industry-relevant constant current of 100 mA g^–1^. A 20% loss in CO_2_ capacity after 50 cycles and a 60% loss after 100 cycles were noted (Figure S16a–g). After 300 cycles, only 20% of the initial uptake capacity was retained. The system maintained a high Coulombic efficiency of 90% (Figure S17). Further limiting the voltage range between −1 and −2 V—a region of predominant redox activity—to minimize parasitic cell degradation at large voltages led to a lower CO_2_ uptake but an improved capacity retention (∼90%) after 100 cycles (Figure S18a–d). These stability issues in ionic liquids motivated us to explore alternate electrolytes.

Having identified key limitations of the capture process in the ionic liquid electrolyte, we replaced this electrolyte with an aqueous 1 M Na_2_SO_4_ electrolyte. Recently, studies have begun to tackle the challenge of performing eCC with quinones in aqueous media.^6,28,29^ Excitingly, a typical eCC cycle (Figure 3a) recorded using AQCOF@CNTs at 100 mA g^–1^ in an aqueous cell exhibited a 4-fold increase in electrochemical CO_2_ uptake compared to ionic liquid cells (IL ∼0.2 mmol g^–1^ and aq. ∼0.8 mmol g^–1^), with the pressure curves closely following the applied voltage. The aqueous cell utilizes ca. 50% of the stored charge to capture CO_2_ with an energy consumption of 37 kJ mol_CO_2__^–1^ at this current density, which is substantially lower than the binding enthalpy of traditional thermal amine processes.^30,31^ Without a voltage hold under pure CO_2_, the uptake showed a negligible change (Figure S19a–c), but the electrical energy consumption of the capture process was further lowered to 31 kJ mol^–1^ (Table S1).

**Figure 3:**
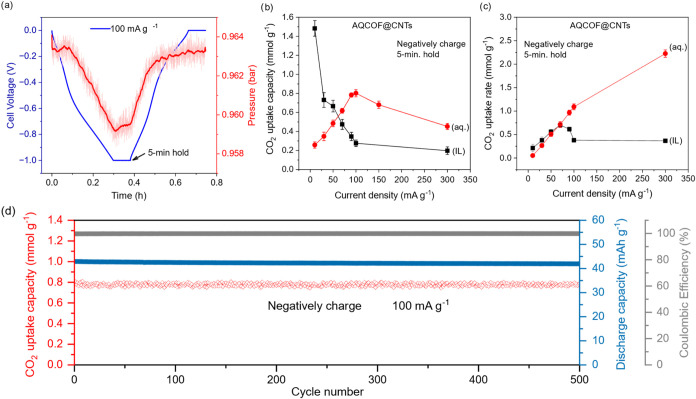
Electrochemical CO_2_ capture performance of the AQCOF@CNT system in 1 M Na_2_SO_4_ as the electrolyte. (a) CO_2_ capture–release cycle recorded at a high current density of 100 mA g_AQCOF@CNTs_^–1^ (the shown data is a smoothed pressure curve moving average every 100 s, red) and a 5 min voltage hold. (b) Comparison of the discharge capacity under CO_2_ and (c) CO_2_ adsorption rate at different current densities measured in [Bmim]­[TFSI] and 1 M Na_2_SO_4_ as the electrolyte. (d) Long cyclic stability in 1 M Na_2_SO_4_ as the electrolyte. In all calculations, the net AQCOF@CNT mass in the electrode was used for the normalization.

Importantly, cyclic voltammetry studies confirmed the presence of quinone redox processes in the studied voltage range (Figure S20), and we further note that our observed electrochemical CO_2_ uptake capacity of ∼0.8 mmol g_AQCOF@CNTs_^–1^ is much larger than that observed in recently reported capacitive CO_2_ capture processes,^15,25,32,33^ indicating that quinone redox drives the electrochemical CO_2_ capture process under aqueous conditions. In our previous work of a cell containing YP80F activated carbon as both the working and counter electrodes with the same aqueous electrolyte, we observed only ∼0.1 mmol g^–1^ CO_2_ adsorption under identical conditions.^24^ Moreover, when we positively charged the cell (0–1 V, i.e., oxidation of the AQCOF@CNT electrode), no CO_2_ uptake was observed, further supporting that the reduction of quinones drives CO_2_ capture (Figure S21).

Electrochemical CO_2_ uptake capacities at different current densities displayed contrasting trends when compared to the ionic liquid system (Figures 3b and S22a–c). The ionic liquid system shows a gradual increase in uptake capacity as the applied current density is decreased, suggesting mass transport limitations at high currents. The aqueous system also shows an increase in the CO_2_ capacity when the current density is decreased from 300 to 100 mA g^–1^; however, this is followed by decreases in capacity as the current density is reduced further (Figure 3b). This suggests that there may be competing mechanisms at play in the aqueous electrolyte, which are discussed further below.

To gauge the potential of the material for practical applications, uptake rates (i.e., CO_2_ capture capacities per unit time) were determined. Figure 3c shows comparable adsorption rates for the IL and aqueous electrolyte systems until 70 mA g^–1^; beyond this point, rates improve further for the aqueous system. Similarly, improvements in Coulombic efficiencies were observed for both the IL and aqueous systems, rising from initial values of 83 and 95% at 5 mA g^–1^ to 98 and 99.6% at 100 mA g^–1^, respectively, indicating a higher reversibility of electrochemical reactions in the aqueous system (Figure S23). The aqueous system also exhibited ultrahigh stability with no obvious charge storage or CO_2_ capacity loss over 500 cycles and with a 99.6% Coulombic efficiency (Figure 3d). The cell is selective toward CO_2_ in a mixture of 85% N_2_:15% CO_2_, (Figure S24), and in an optimized cycle (100 mA g^–1^ charging current, no voltage holds, 0 to −0.8 V cell voltage window), an exceptionally low electrical energy consumption of 28 kJ mol^–1^ and superior adsorption capacity and adsorption rates were realized (Figure S25). However, measurements under 15% O_2_ show only 0.34 mmol g^–1^ CO_2_ uptake with a decreased Coulombic efficiency of 91.2% (Figure S26a–d). Future work should address this challenge by developing redox-active COFs with more positive redox potentials and using stabilizing intermolecular interactions to break the unfavorable scaling relationship between the redox potential and the CO_2_ binding energy.^34^

Postcycling analysis of COF@CNT electrode films shows structural changes after extended electrochemical use. In free-standing COF films, COF diffraction peaks are partially obscured by the polytetrafluoroethylene (PTFE) binder and CNTs, making it difficult to assess changes in crystallinity (Figure S27a–c). Films cycled in ionic liquid electrolytes show a reduced PTFE peak intensity, indicating a possible breakdown under high reductive currents. This degradation may cause decomposition products to accumulate within the porous structure and contribute to gradual capacity loss in the ionic liquid cell (Figures S16 and S17). Scanning electron microscopy (SEM) images of used electrodes reveal surface damage in the IL sample, and the comparatively aqueous sample largely remains morphologically stable (Figure S27d–f). These results indicate that the electrode films undergo mechanical degradation during extended cycling.

To try to better understand the underlying processes involved in the capture of CO_2_ in aqueous cells, we explored different voltage regimes. While working in a 0 to −0.5 V window, a CO_2_ uptake of ∼0.2 mmol g^–1^ was noted and was relatively constant at different current densities (Figure S28a). This behavior is similar to the trend seen in recent supercapacitive swing adsorption experiments.^32,33^ In contrast, CO_2_ uptake in the −0.5 to −1 V window showed a progressive increase as a function of current until 100 mA g^–1^ where it reaches 0.4 mmol g^–1^ (Figure S28b). We note the possibility of both the “direct redox capture” (Figure 1b) of CO_2_ by the reduced anthraquinone units as well as the occurrence of pH swing (Figure 1c) in the aqueous electrolyte used here, which is discussed in more detail below.^6,35^

### CO_2_ Capture Mechanism Study

Evidence for direct redox capture (Figure 1b) by reduced AQCOF samples was obtained using a chemical reduction approach (SI Methods Section II, Figures S30–S42). In contrast to the parent AQCOF, the chemically reduced AQCOF exhibited pronounced CO_2_ chemisorption in CO_2_ adsorption isotherms and isobars (Figures S34 and S38). In the Fourier transform infrared (FTIR) spectra (Figures 4a and S35), the disappearance of the C=O stretch of the quinone following chemical reduction and the appearance of a new peak at ∼1650 cm^–1^ after CO_2_ dosing are consistent with carbonate formation and closely match the spectral features reported for anthraquinone–CO_2_ adducts in molecular systems.^36,37^ These findings support the proposed mechanism of covalent CO_2_ binding at the electrochemically reduced quinone sites. The CO_2_ uptake behavior of COF@CNTs in an ionic liquid electrolyte under different voltage windows (Figures 2d and S11) also indicated that CO_2_ uptake is initiated via quinone redox processes, consistent with the “direct capture” route to form anthraquinone–CO_2_ adducts.

**Figure 4:**
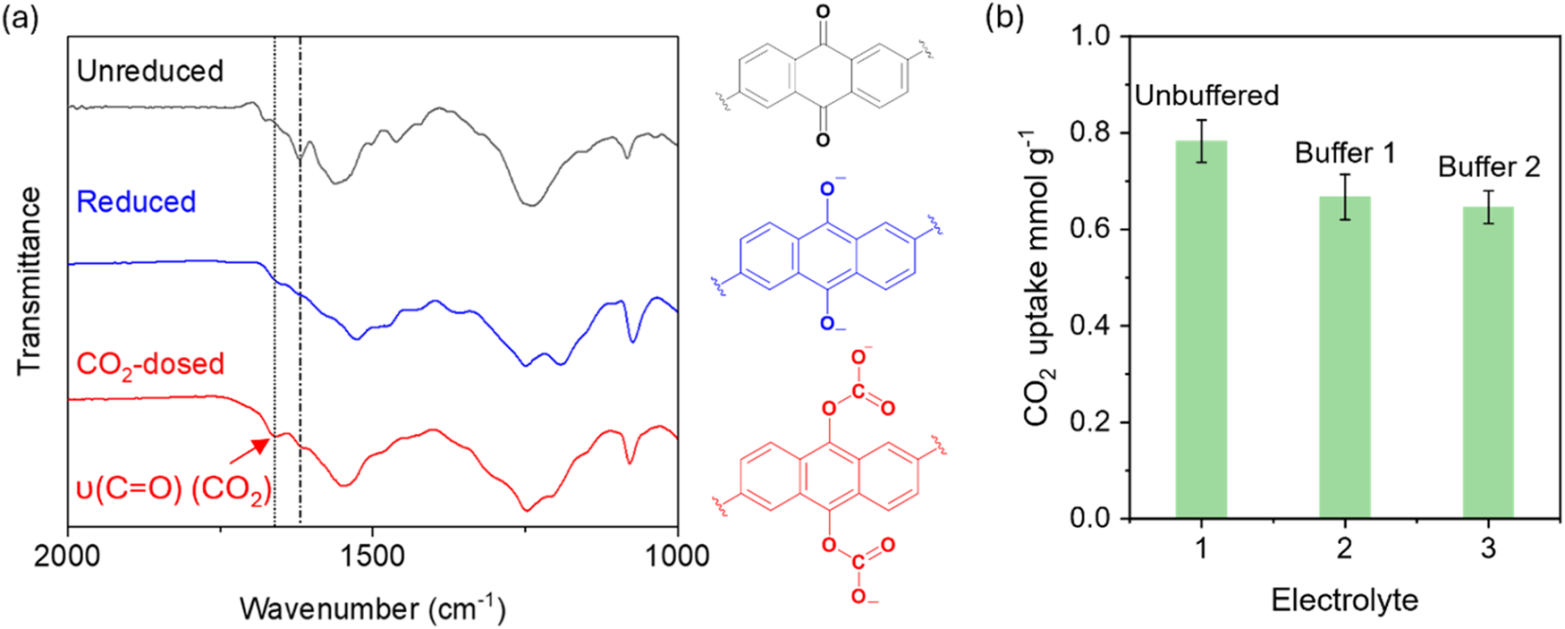
(a) FTIR spectra of the unreduced, chemically reduced, and CO_2_-dosed AQCOF material. (b) CO_2_ uptake of AQCOF@CNTs in the unbuffered 1 M Na_2_SO_4_ electrolyte and in phosphate-buffered electrolytes: Buffer 1 (0.1 M NaH_2_PO_4_/Na_2_HPO_4_ with 0.9 M Na_2_SO_4_) and Buffer 2 (0.5 M NaH_2_PO_4_/Na_2_HPO_4_ with 0.5 M Na_2_SO_4_).

To assess the possible pH-driven CO_2_ uptake in aqueous systems (Figure 1c), additional measurements were conducted in 0.1 and 0.5 M phosphate-buffered electrolytes, with all other conditions unchanged and a total ∼2 M Na^+^ concentration. These concentrations assume that all of the CO_2_ uptake occurs via pH swing and exceeds those needed to suppress CO_2_ uptake equilibria involving CO_2_, HCO_3_^–^, and CO_3_^2–^ species. Results show a ∼(0.1–0.12) ± 0.03 mmol g^–1^ decrease in CO_2_ uptake under buffered conditions. This suggests that some of the apparent capacity in the unbuffered electrolyte comes from a surface-driven pH change, while most of the uptake is due to direct electrochemical capture by reduced quinone (Figures 4b and S29a–c). We tentatively propose that the pH swing mechanism might be responsible for the decrease in electrochemical CO_2_ uptake seen at low current densities (Figure 3b) due to neutralization of pH changes by mass transport processes (e.g., H_3_O^+^ diffusion) when charging slowly. Identification of the exact species involved and full clarification of the electrochemical CO_2_ capture mechanisms in this system require further study.

## Conclusions

In conclusion, we have demonstrated the first example of the use of a covalent organic framework for electrochemical CO_2_ capture. Electrochemical reduction of the anthraquinone units in the COF led to the electrochemical capture of CO_2_ in electrochemical cells with both ionic liquid and aqueous electrolytes. While cells with ionic liquid electrolytes showed poor kinetic behavior and low stability, cells with aqueous electrolytes showed a greatly improved performance, with higher CO_2_ uptake rates and excellent long-term stability. Our measurements also indicate a direct redox capture mechanism in ionic liquid electrolytes and both direct redox capture and pH swing mechanisms in aqueous electrolytes. Ultimately, this study opens a new material class for electrochemical CO_2_ capture and may lead to further performance improvements as well as a new understanding of how to control the thermodynamics and kinetics of this important process. Future work should further explore the capture mechanism in these materials, and life cycle assessments and techno-economic analyses should be used to evaluate the sustainability and economics of the use of COFs for electrochemical separations.

## Supplementary Material



## Data Availability

All data are available in the main text or in the Supporting Information.

## Notes

Experimental details on synthesis; characterization of the materials; preparation of films, electrodes, and electrochemical cells; and details of the gas manifold and gas dosing setup, electrochemical gas cell device, CV measurements, electrochemical CO_2_ capture measurements, and chemical reduction and CO_2_ capture measurements (PDF)

## a

Institute for Technical Chemistry and Environmental Chemistry, Friedrich-Schiller-University, Philosophenweg 7a, 07743 Jena, Germany
