# Supplementary material for: Electrochemical CO2 Capture by a Quinone-Based Covalent Organic Framework
Source: J Am Chem Soc. 2025 Dec 9;147(51):47036–43. doi: 10.1021/jacs.5c12304 (PMC12751002; doi:10.1021/jacs.5c12304)
Supplement: Supplementary file 1 [file ja5c12304_si_001.pdf]

Supporting Information

Electrochemical CO_2 Capture by a Quinone-Based Covalent Organic Framework

Muhammad Abdullah Khan [a, b]*, *Zhen Xu,* [a, j] *Muhammad Muzammil* [b], *Samuel Bird* [a], *Monica Munawar* [b], *Fariah Salam* [b], *Niamh A. Hartley* [a], *Jack Taylor* [a], *Kamran Amin* [c], *Jianheng Ling* [d], *Henry R. N. B. Enninful* [a, e], *Naveed Zafar Ali* [f], *Kai Hetze* [g, h], *Sijia Cao* [i], *Yan Lu* [i, g, h], *Zhixiang Wei* [c], *Martin Oschatz* [g, h], *Phillip J. Milner* [d], and *Alexander C. Forse* [a]*

[a] Yusuf Hamied Department of Chemistry, University of Cambridge, Lensfield Road, Cambridge CB2 1EW, U.K

[b] Renewable Energy Advancement Laboratory, Department of Environmental Sciences, Quaid-i-Azam University Islamabad, 45320, Pakistan

[c] CAS Key Laboratory of Nanosystems and Hierarchical Fabrication, National Center for Nanoscience and Technology, Chinese Academy of Sciences, Beijing, 100190, P. R. China

[d] Department of Chemistry and Chemical Biology, Cornell University, Ithaca, New York 14850, United States

[e] Felix Bloch Institute for Solid State Physics, Faculty of Physics and Earth Sciences, Leipzig University, Linnéstraße 504103, Leipzig, Germany

[f] National Centre for Physics, Quaid-i-Azam University, Islamabad 44000, Pakistan

[g] Institute for Technical Chemistry and Environmental Chemistry, Friedrich-Schiller-University Jena, Philosophenweg 7a, 07743, Jena, Germany

[h] Helmholtz Institute for Polymers in Energy Applications Jena, Lessingstraße 12–14, 07743 Jena, Germany

[i] Helmholtz-Zentrum Berlin für Materialien und Energie, Hahn-Meitner-Platz 1, 14109, Berlin, Germany

[j] Department of Materials and Henry Royce Institute, University of Manchester, Manchester M13 9PL, U.K.

*Corresponding authors

Email:

makhan@qau.edu.pk (Muhammad Abdullah Khan); acf50@cam.ac.uk (Alexander C. Forse)

SECTION I. Electrochemical Reduction and CO_2 capture

Materials and Methods

To synthesize anthraquinone-based covalent organic framework (AQCOF), 2,6-diaminoanthraquinone (DAAQ), 3,5-triformylphloroglucinol (TFP), dimethylacetamide (DMAc), mesitylene, acetic acid, polytetrafluoroethylene (PTFE) 60% dispersion in water were procured from Sigma-Aldrich. In addition, *N, N*-dimethylformamide (99%) was purchased from Alfa Aesar, while acetone and ethanol (>99%) were procured from VWR Chemicals. Acetylene black was procured from Thermo Scientific Carbon nanotubes, High purity single walled carbon nanotubes (CNTs) were purchased from TimesNano. The outer diameter was less than 2 nm and purity was greater than 95 % as mentioned by supplier. These CNTs were further washed with water vapors and heat treated at 900 ºC in Ar environment to remove impurities further. Microporous YP-80F powder was purchased from Kuraray. 1-Butyl-3-methylimidazolium bis(trifluoromethyl sulfonyl)imide (99%) was purchased from Iolitec. It was then dried under vacuum for 6 days and stored in an N_2 glovebox.

1.1 Synthesis

Synthesis of anthraquinone covalent organic framework (AQCOF)

For the synthesis of AQCOF a reported procedure was followed.[1,2] A 10 mL Pyrex tube was charged with 1,3,5-triformylphloroglucinol (TFP) (20 mg, 0.095 mmol), 2,6-diaminoanthraquinone (DAAQ) (34 mg, 0.142 mmol), 0.9 mL dimethylacetamide (DMAc) and 0.3 mL mesitylene. The mixture was sonicated for 20 minutes and 50 µL of 6M acetic acid was added. The tube was subjected to freeze pump thaw cycles thrice, vacuumed, and placed in an oven at 120 ºC for three days. The obtained dark red powder was washed with 30 mL of DMF 5 times and solvent exchanged with 30 mL of acetone twice. Then dried at 180 ºC in a vacuum oven for 24 hrs. The final AQCOF (reddish brown in color) yield was about 71%. A summarized synthesized process is presented in Figure S1.

FTIR: 1658w, 1615w, 1555s, 1240s, 1080, 972m, 890s,813m, 744m, 572, 496 cm^{-1} (Figure S4e)

Solid State NMR: δ 108 s, 118 w, 123w, 129 s, 135s, 144s 181 m, ppm (Figure S4f)

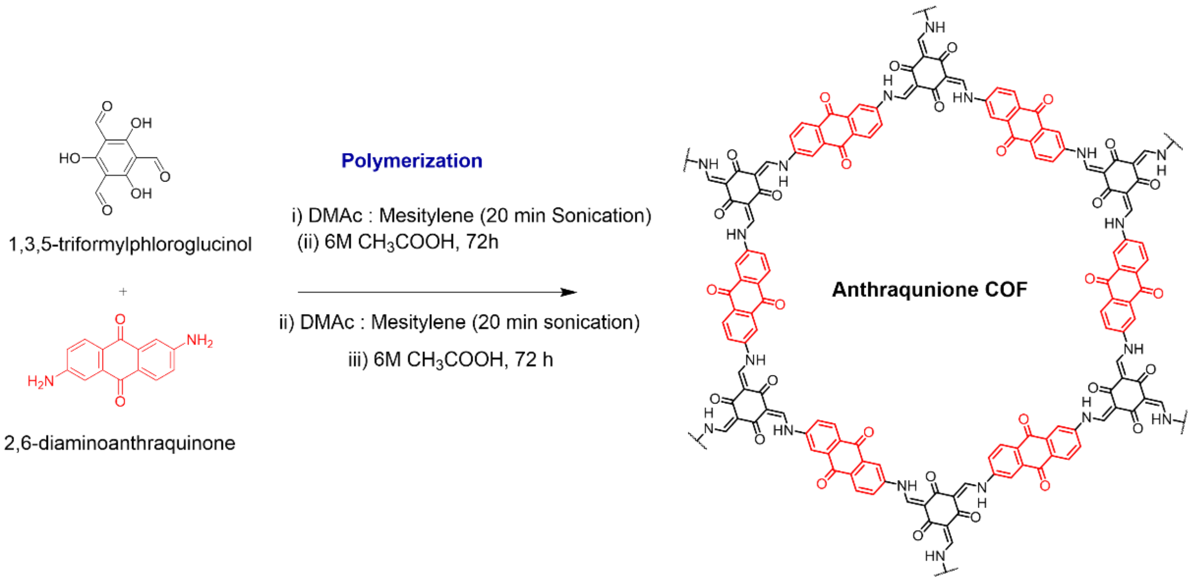

Figure S1. Schematic representation of AQCOF synthesis.

Synthesis of AQCOF@CNTs

AQ-COF@CNTs was synthesized in an *in-situ* polymerization process (Figure S2).[1,2] In the first step, 3 mg of CNTs and 34 mg DAAQ were sonicated in ethanol for 2 hours followed by drying at 50 ºC under vacuum to obtain a well dispersed CNTs/DAAQ powder. The powder was then transferred to a reaction flask containing 20 mg TFP, DMAc, and 0.4 mL mesitylene were added as solvent. The mixture was sonicated for 20 minutes and 50 µL of 6M acetic acid was added. All other reaction steps were the same as mentioned above. The material with 7% CNTs (mass before reaction) was prepared and characterized. The ratio of CNTs was calculated based on the total mass of the reactants and CNTs combined, e.g., a sample with a total mass of 57 mg containing 4 mg of CNTs is labeled as AQ COF@CNTs (7%).

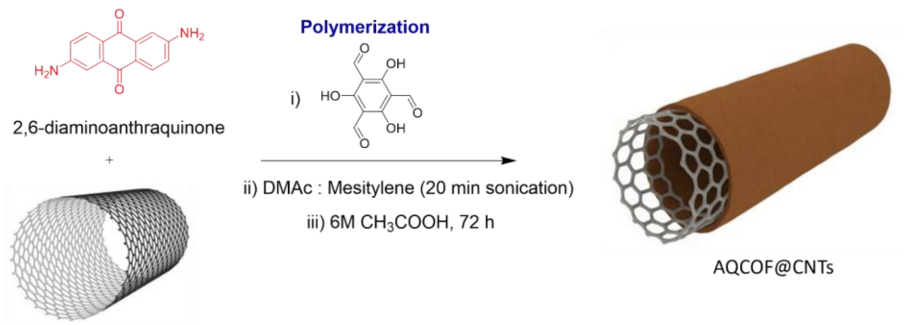

Figure S2. Schematics for the synthesis of the AQCOF@CNTs.

2 Preparation of electrodes

2.1 Electrode films

Freestanding electrode films were prepared following a literature method with modifications[3,4] . First, 35 mg of AQCOF powder and 10 mg of acetylene black carbon were mixed with ethanol (ca. 3 mL) to make a loose slurry on a watch glass. Then, 5 mg of polytetrafluoroethylene (PTFE) dispersion in water were added and the mixture was stirred vigorously by hand for 30 minutes under ambient conditions. As the ethanol evaporated, the film gradually formed, which was kneaded with a spatula for 15 minutes to attain consistency and enhanced mechanical strength. Afterward, the material was transferred to a glass slab and rolled into a freestanding film using an aluminum roller (thickness 0.25 mm). The film was then dried in vacuum oven at 90 ºC for at least 24 hours to remove solvent traces. The final composition of the film consisted of 70 wt% AQCOF, 20 wt% Acetylene Black Carbon, and 10 wt% PTFE. The same procedure was followed to prepare AQCOF@CNTs freestanding films. All the films maintaining a thickness of *ca.* 0.25 mm, were cut and stored for further use.

Likewise, YP-80F films were prepared adopting the procedure used for COF films, where final composition of the film electrode was 95 wt% YP-80F and 5 wt% PTFE.

Preparation of electrodes

Working electrodes were cut from freestanding AQCOF, or AQCOF@CNTs composites and YP-80F films with a mass ranging between 5–10 mg cm^{-2}. The weight ratio between the negative (AQCOF or AQCOF@CNTs) and positive (YP80F) electrode was kept at 1: >2.5, respectively to ensure excess charging capacity on the YP-80F electrode (relative to the AQCOF electrode). In this way, the potential on the working AQCOF electrode limits the cell's stable working voltage range. To separate the working and counter electrodes, a Whatman glass microfiber filter (GF/A) was used, which was dried *in vacuo* at 100 ºC for 24 h before use. Two stainless steel spacer disks and one spring were used in each cell to maintain consistent pressure.

Electrochemical cells, containing 1-Butyl-3-methylimidazolium bis(tri-fluoromethyl sulfonyl)imide ([Bmim][TFSI]) ionic liquid (200 µL) as an electrolyte, were assembled inside a glovebox under nitrogen (N_2) atmosphere. The cells were sealed within the glovebox using a Compact Hydraulic Coin Cell Crimper (Cambridge Energy Solutions) and placed in a gas manifold (shown in Figure S3b) before being removed for testing.

To construct electrochemical cells containing aqueous electrolyte, the same procedure outside the glovebox was followed. Where 1 M Na_2SO_4 (200 µL) was added as an electrolyte while the rest of the components and steps remained the same.

2.3 Composition and configuration

A typical electrochemical cell composition and configuration include a meshed top case (SS316 CR2032, Cambridge Energy Solutions), a working electrode (either AQCOF or AQCOF@CNTs), two GF/A

separators (Whatman, diameter: 0.20 mm), electrolyte immersed electrode (YP80F), 200 µL of 1-butyl-3-methylimidazolium bis(trifluoromethylsulfonyl)imide ([Bmim][TFSI]) ionic liquid, two 0.5 mm stainless steel spacers, one conical spring, and a bottom case (Figure S3a). Following assembly, the components, 'AQCOF@CNTs/[Bmim][TFSI] electrolyte/YP80F' were securely pressed within the meshed coin cell, resulting in a final cell thickness of 3.2 mm.

For aqueous cells, all the components were same except 200 µL of 1 M Na_2SO_4 electrolyte was used instead of IL electrolyte.

2.4 Dosing protocol for electrochemical gas cell

The CO_2 uptake experiments were conducted using our customized gas manifold shown in Figure S3. The image is modified from our previous report [4]. Where electrochemical cells in a gas manifold were placed meshed side facing the gas reservoir to introduce gases CO_2 (99.80% purity, BOC), N_2 (99.998% purity, BOC) or gas mixture.

To dose the electrochemical cell with CO_2, the gas manifold was connected to the dosing setup (Figure S3). For ionic liquid (IL)-based electrochemical cells, the nitrogen (N_2, 1 bar) initially present in the cell headspace was replaced with carbon dioxide (CO_2) through a controlled series of steps. First, with valve G2 on the gas manifold closed, the dosing line between G2 and valves 1–4 was fully evacuated. Next, valve 3 (connected to the CO_2 source) and valve 1 (connected to the vacuum pump) were closed, and valve G2 was opened. This step increased the pressure in the dosing line to approximately 450 mbar, thereby preventing the electrochemical cell from being exposed to a full vacuum. Following this, valve G2 was closed again. The dosing line was evacuated and then filled with pure CO_2 until the pressure reached 1.2 bar. Valve G2 was reopened, allowing the pressure to equilibrate to approximately 1 bar. Valve G2 was then closed once more. The cycle—closing valve G2, evacuating the line, and refilling with CO_2—was repeated 10 times to achieve ≈100% CO_2 saturation in the headspace of the cell. Throughout the process, valve G1 remained open and was only closed prior to disconnecting the gas manifold from the dosing line. The same procedure was used for introducing other gases into the cell.

For aqueous electrochemical cells, the procedure was identical, except that these cells were prepared on the laboratory bench (rather than in a glovebox) and initially contained air in the headspace.

4.1 Dosing protocol for O_2 into the Electrochemical Cell

For the introduction of O_2 into the electrochemical device, the cell was first fully dosed with 100% CO_2 following the protocol described above. Afterwards, the gas line between valve G2 and the pump was evacuated, reducing the pressure to approximately 0.5 bar, corresponding to about 18 mL of gas in the reservoir. Valve G2 was then closed, and the gas line was re-evacuated before

introducing 1 bar of air. Subsequently, valve G2 was opened to allow the air (1 bar, 30 mL) to enter the gas reservoir, bringing the total pressure to approximately 0.8 bar and yielding a gas composition of ~20% CO_2, 15% O_2, and 65% N_2 within the manifold. Throughout the procedure, valve G1 remained open and was only closed immediately before disconnecting the gas manifold from the dosing line.

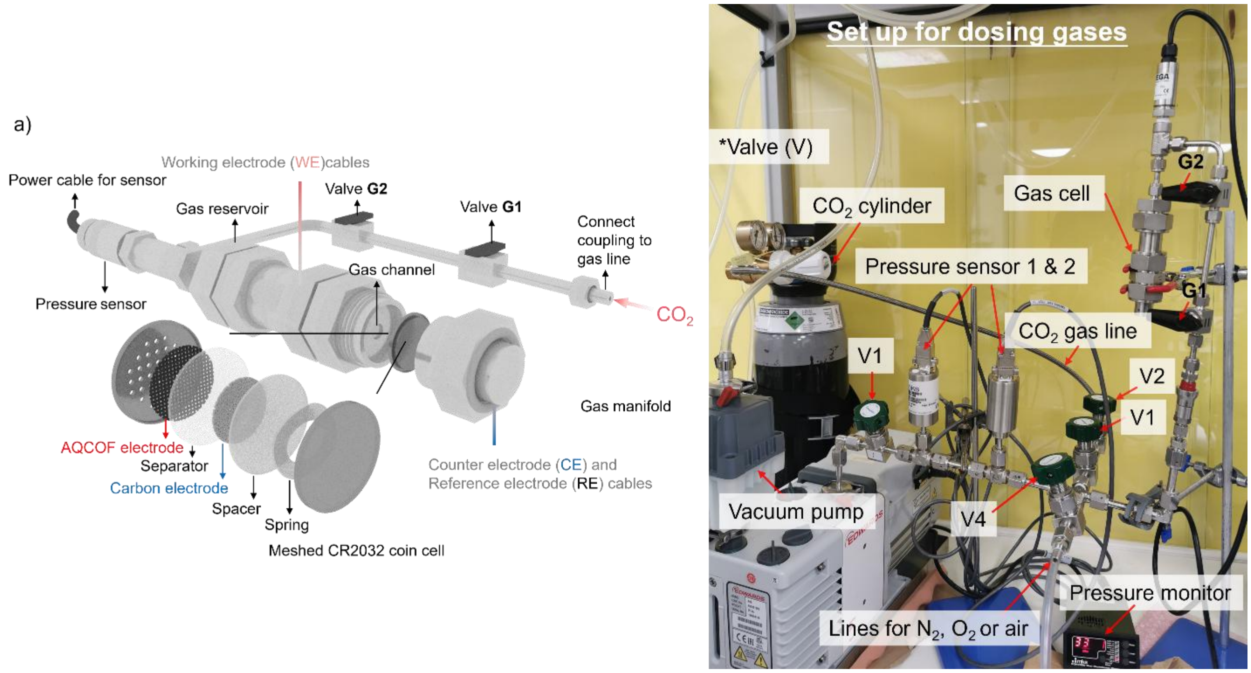

Figure S3. Schematic of coin cell, gas manifold and dosing setup for electrochemical CO_2 uptake measurements. (a) Composition and configuration of the meshed coin-type electrochemical cell used for gas uptake and energy storage studies along withs schematic of our customized gas manifold system, designed to hold meshed electrochemical cell for controlled CO_2 uptake measurements. Modified from Ref. [4]. (b) Photograph of the complete laboratory setup showing the gas manifold connected to the electrochemical cell, used for introducing gas mixtures into the electrochemical cell during gas uptake experiments.

2.5. Electrochemical measurements

2.5.1 Electrochemical stability window of the ionic liquid electrolyte

The electrochemical stability window of the ionic liquid electrolyte for CO_2 capture experiments was determined using the cell configuration described in section 2.3 without COF or carbon electrodes. The cell voltage was scanned from 0 to –3.5 V, and an operating range of 0 to –2.5 V was selected for subsequent electrochemical measurements. Within this range, the ionic liquid exhibited only a very small faradaic current, confirming its electrochemical stability under the applied conditions.

2.5.2 CO_2 uptake measurements

The electrochemical testing of the gas cells, including galvanostatic charge and discharge measurements (GCD) and cyclic voltammetry (CV), was performed using a potentiostat (VSP-3e or VMP-3e, Biologic). CO_2 uptake, monitored as pressure a change resulting from gas capture and release, was measured using a high-sensitivity pressure sensor (Omega PX309-030A5V) integrated with the potentiostat for simultaneous acquisition of electrochemical and pressure data. Pressure sensors are suitable for monitoring the overall pressure change, capturing all CO_2-related processes (capture, release, and any irreversible losses) without relying on optical path or calibration drift. They work regardless of gas humidity, cell design, or impurities and are particularly suited for our proof-of-concept measurements. The pressure sensor has sensitivity with a noise of 0.1 mbar, and the signal-to-noise ratio over 5. In measurement to minimize random pressure noise, the pressure data were averaged (moving average) every 100 seconds. The accuracy and reliability of the pressure readings were further validated using two additional pressure sensors (MKS PDR2000 Dual Capacitance Manometers) with a higher sensitivity of 0.01 mbar.

2.5.3 CO_2 uptake measurements in buffered electrolyte

To evaluate the possible contribution of pH-driven CO_2 uptake, electrochemical measurements were performed in phosphate-buffered electrolytes (containing NaH_2PO_4 and Na_2HPO_4 salts) with buffer concentrations of 0.1 M and 0.5 M, along with 0.9 M and 0.5 M Na_2SO_5, respectively. Both buffer concentrations were sufficient to neutralize any local pH change and to ensure that protons were not available for CO_2 uptake via the pH-swing pathway. All other experimental conditions and cell configurations were identical to those used in the standard setup.

Table 1. Chemical details for preparation of the puffer solution prepared in 10 mL

**Electrolyte**	**Na_2SO_4 (anhydrous)**	**NaH_2PO_4**	**Na_2HPO_4**	**Notes**
0.10 M buffer	1.42 g	0.60 g (anhydrous)	0.71 g (anhydrous)	pH ~ 7.2
0.50 M buffer	1.42 g	0.30 g (anhydrous)	0.35 g (anhydrous)	pH ~ 7.2

All the electrochemical CO_2 capture tests were then carried out under identical conditions to those using unbuffered 1.0 M Na_2SO_4 electrolyte.

2.6 Analysis of Spent Samples

After the long cycling tests, post-cycling characterization was performed on the spent electrode films. Samples from the ionic-liquid–based electrolyte were collected from cells that were disassembled in a N_2-filled glovebox. The electrodes were gently rinsed with anhydrous acetonitrile (three times), followed by anhydrous isopropanol (three times), and then vacuum-dried at 70 ºC overnight. Films recovered from aqueous cells were rinsed with ultrapure deionized water (five times) and subsequently dried in a vacuum oven at 70 ºC overnight.

3. Characterization

Powder X-ray diffraction data were collected on a Malvern Panalytical Empyrean instrument, equipped with an X'celerator Scientific detector using non-monochromatic CuKα radiation (λ= 1.5418Å). The sample was mounted on a silicon zero background plate and measured in reflection geometry with sample spinning. The data was collected at room temperature over a 2θ range of 2 - 40 °, with an effective step size of 0.02 ° and a total collection time of 1 hour. XRD of spent electrodes was conducted using a Bruker D8 Advance with CuKa radiation and a LynxEye XE-T 1D detector. Diffractograms were recorded over 2θ range of 5- 70°, with a step size of 0.02° and a step time of 1 second. Micromeritics ASAP 2420 accelerated surface area and porosimeter system was used to analyze the BET surface area and pore size distribution. All the samples were outgassed at 473 K for 6 h before the N_2 adsorption measurements. N_2 gas adsorbate was used with 2 min equilibration times at each point. The diameter volumetric distribution and mean pore diameter was determined by the Barrett– Joyner–Halenda (BJH) method, from the adsorption branch of isotherm, and pore size distributions were obtained using the quenched solid density functional theory (QSDFT) and slit pore model (*1*). Solid-state carbon-13 NMR (^{13}C NMR) spectra were acquired using a JNM-ECZ600R spectrometer, operating at a spinning rate of 12 kHz and a relaxation delay of 3 seconds. Fourier transform infrared spectroscopy with attenuated total reflection (FTIR-ATR) was performed using a Spectrum One Perkin-Elmer instrument over a range of 400–4000 cm^{-1}. Transmission electron microscopy (TEM) was performed on JEOL JEM-2100 (JEOL GmbH, Eching, Germany) operated at 200 kV. COF samples for TEM were sonicated in methanol for 5 min and a 5 μL of sample dispersion was applied to Holey carbon-coated copper TEM grids (200 mesh, Science Services) and subsequently dried under a fume hood. Scanning electron microscopy (SEM) analysis was conducted using a Zeiss Merlin electron microscope. An accelerating voltage of 4 kV was applied for image acquisition. The pristine COF samples were mounted on carbon tape and coated with a thin layer of gold to enhance electrical conductivity during SEM measurements. The surface chemistry of AQCOF@CNTS materials was characterized using X-ray photoelectron spectroscopy using a Thermo Scientific K-Alpha XPS instrument equipped with a micro-focused monochromatic Al-Kα X-ray source. Prior to the analysis by X-ray Photoelectron Spectroscopy (XPS), the samples were degassed in a high vacuum (< 5×10^{-7} bar) for 90 minutes. Survey scans were conducted with a pass energy of 200 eV, a step size of 1 eV, and dwell times of 200 ms (10 ms × 20 scans). The data were analyzed using Avantage software. Data acquisition and analysis was done with Avantage software, Igor Pro. Peak spectra fitting was performed using a Gaussian-Lorentzian line shape after applying shirley background corrections.

4. Calculations

The specific gravimetric CO_2 adsorption capacity (C_{CO2}, $mmol_{CO2}$ kg^{-1}) was calculated by determining the difference in gas amount (Δn, mol) between the average of two maximum peaks (n_{max_1} and n_{max_2}) and the minimum peak (n_{min}) in the reservoir and normalizing this value by the active mass (AQCOF or AQCOF@CNTs mass) of the working electrode. To account for any irreversible pressure change, due to side reactions, the average of two minimum points was used to ensure reliability in estimation of adsorption capacities. Gas amounts were derived from pressure sensor data (moving average every 100 seconds), converted using the ideal gas law:

1. $$n = \frac{PV}{RT}$$

2. $$\Delta n = \frac{n_{max_1} + n_{max_2}}{2} - n_{min}$$

3. $$C_{CO_2} = \frac{\Delta n}{m} \times 10^6$$

Here, P is pressure (Pa), V is volume (m^3), T is temperature (K), R is the ideal gas constant (8.31451 m^3 Pa mol^{-1} K^{-1}), and n is the number of moles of gas. In each CO_2 adsorption–desorption cycle, n_{max_1} and n_{max_2} represent the two maximum values of the moles of gas in the chamber, and n_{min} corresponds to the minimum of the moles of gas in the gas chamber for a given cycle. The reported adsorption capacity represents the average over multiple (at least five) CO_2 adsorption–desorption cycles, with associated error estimated using a 95% confidence interval via the Student's t-test.

The volume of the gas reservoir (V_1, mL) in the gas cell was determined during the CO_2 dosing process, based on pressure measurements of the added gas, using Boyle's Law ($P_1V_1 = P_2V_2$). For each gas cell, the volume of the pipe between two valves (A, mL) had been previously calibrated. Initially, this section was evacuated to remove residual gas, and the pressure (P_1, bar) of the gas in the main reservoir was then recorded, representing a gas quantity proportional to P_1V_1. Next, the valve to the evacuated volume (A, mL) was opened, allowing the gas to fill the additional space. Since the total amount of gas remained same (therefore, $P_1V_1 = P_2V_2$) but the pressure decreased to P_2 (bar). As the V_2 (mL) = V_1 (mL) + A (mL), the reservoir volume was then calculated using the equation:

4. $$V_1 = \frac{P_2 A}{P_1 - P_2}$$

This procedure also served as a leak test for the gas cell. In the absence of leaks, the final pressure (P_2) remained stable and below 1 bar. A rising pressure would indicate a leak, invalidating the volume measurement.

Adsorption rate and energy consumption:

The specific electrical energy consumption (E, kJ mol CO_2^{-1}) and adsorption rate (RCO_2, mmolCO_2 kg^{-1} h^{-1}) were calculated using equations, as follows:

5. $$E = \frac{E_{in} - E_{out}}{c_{CO_2}} \times 10^3$$

6. $$R_{CO_2} = \frac{c_{CO_2}}{t_{charging} \times 3600}$$

Here, E_{in} and E_{out} out represent the total electrical energy input and output, respectively, C_{CO2} denotes the amount of CO_2 adsorbed or captured (in mol) and $t_{charging}$ is the total charging duration in seconds, including any voltage hold step.

Coulombic Efficiency

Coulombic efficiency (CE, %) was calculated to assess the reversibility of charge storage, using the following equation:

7. $$CE = \frac{\int_{t_3}^{t_4} I(t)\, dt}{\int_{t_1}^{t_2} I(t)\, dt} \times 100\%$$

Here, *I(t)* (A) represent the current that changes over time. The variables t_1 and t_2 (s) denote the start and end times of the charging process, respectively, while t_3 and t_4 (s) correspond to the start and end times of the discharging process.

Discharge Capacity

The discharge capacity of the electrode was calculated from GCD (galvanostatic charge-discharge) measurements according to equation

8. $$C_{electrode} = C\frac{Q}{m}$$

Where C (mAh g^{-1}) is the capacity of the electrode, Q is the total charge (mAh), calculated by integrating the current overtime during the discharge process, i.e., $Q = \int I(t)dt$, and m is the mass of the active material

of the electrode (in grams). This formula calculates the charge stored or released, then normalizes it by the mass of the electrode material to obtain the capacity in terms of charge per unit mass.

CO_2 to electron utilization

9. $$\mathrm{CO_2\ to\ electron\ utlization} = \frac{\mathrm{Moles\ of\ CO_2\ captured}}{\mathrm{Moles\ of\ electrons\ transferred}}$$

$$R_{CO_2} = \frac{n_{CO_2}}{Q/F}$$

Where n_{CO2} is moles of CO_2 captured, Q is total charge passed (in coulombs) and F is Faraday constant = 96485 C mol^{-1} (charge per mole of electrons)

5. Figures and Tables

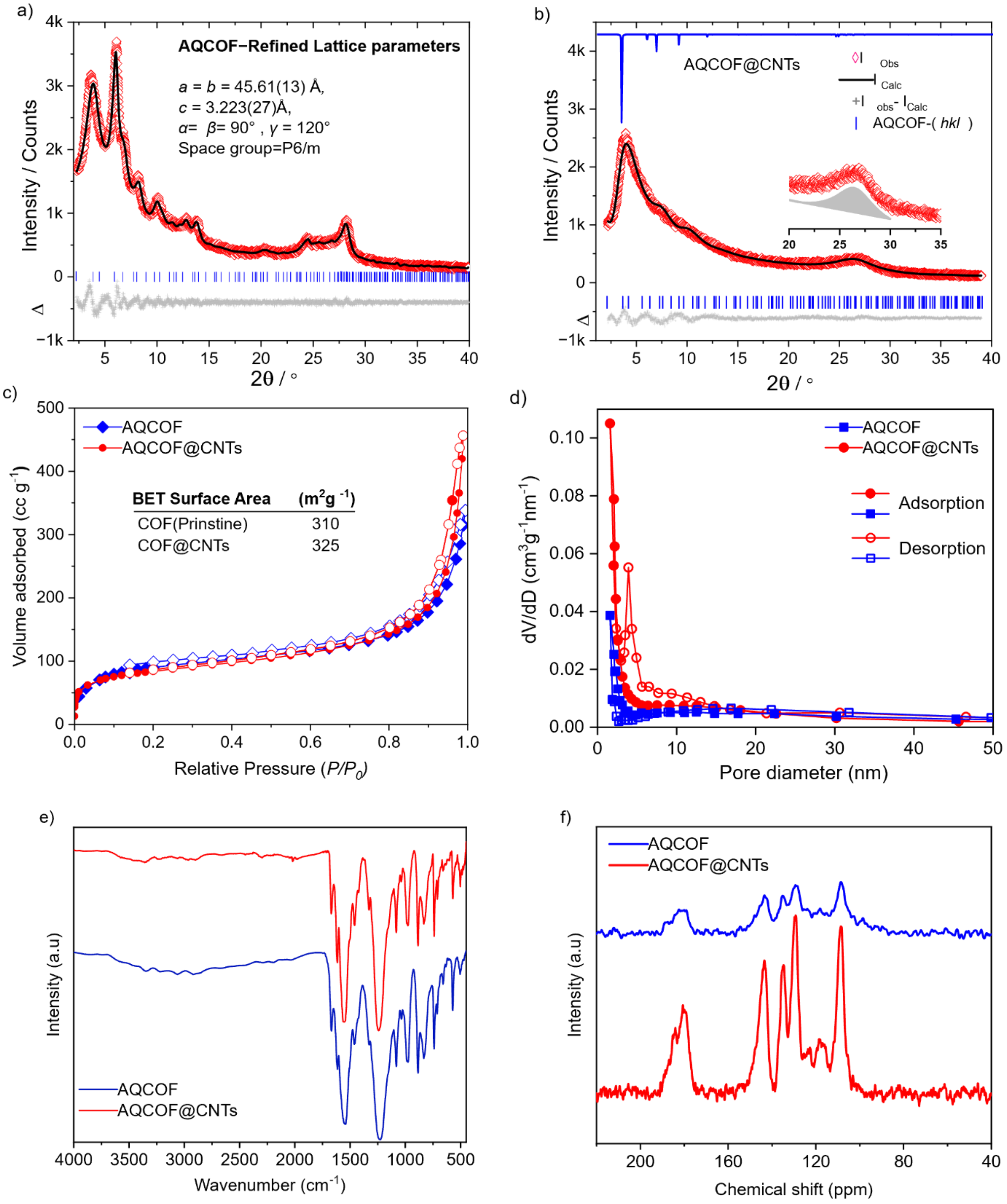

Figure S4. Structural characterization of COF materials. PXRD patterns of (a) AQCOF and (b) AQCOF@CNTs composite material. Le Bail fitting of experimental powder XRD data of (a) AQCOF and (b) AQCOF@CNTs composite at 298 K, with refined lattice parameter retrieved from hexagonal (P6/m) structure (ref. [5]) and corresponding simulated diffraction pattern model shown inverted in Fig.S4(a). The

inset in Figure S4(b) shows the bump attributed to CNT contribution. In the AQCOF@CNTs composite, the characteristic peaks of the covalent organic framework (COF) are retained, indicating the structural integrity of the COF component. The observed peak broadening, however, can be attributed to the constrained growth of COF domains on the carbon nanotube (CNT) surfaces, which significantly limits their crystallite size. These and all other characterization results are consistent with previously reported findings in the literature (c) N_2 adsorption–desorption isotherms and (d) corresponding pore size distributions for AQCOF and AQCOF@CNTs. (e) Fourier-transform infrared (FTIR) spectra confirming the presence of characteristic functional groups and (f) solid-state ^{13}C NMR spectra of AQCOF and AQCOF@CNTs, respectively.

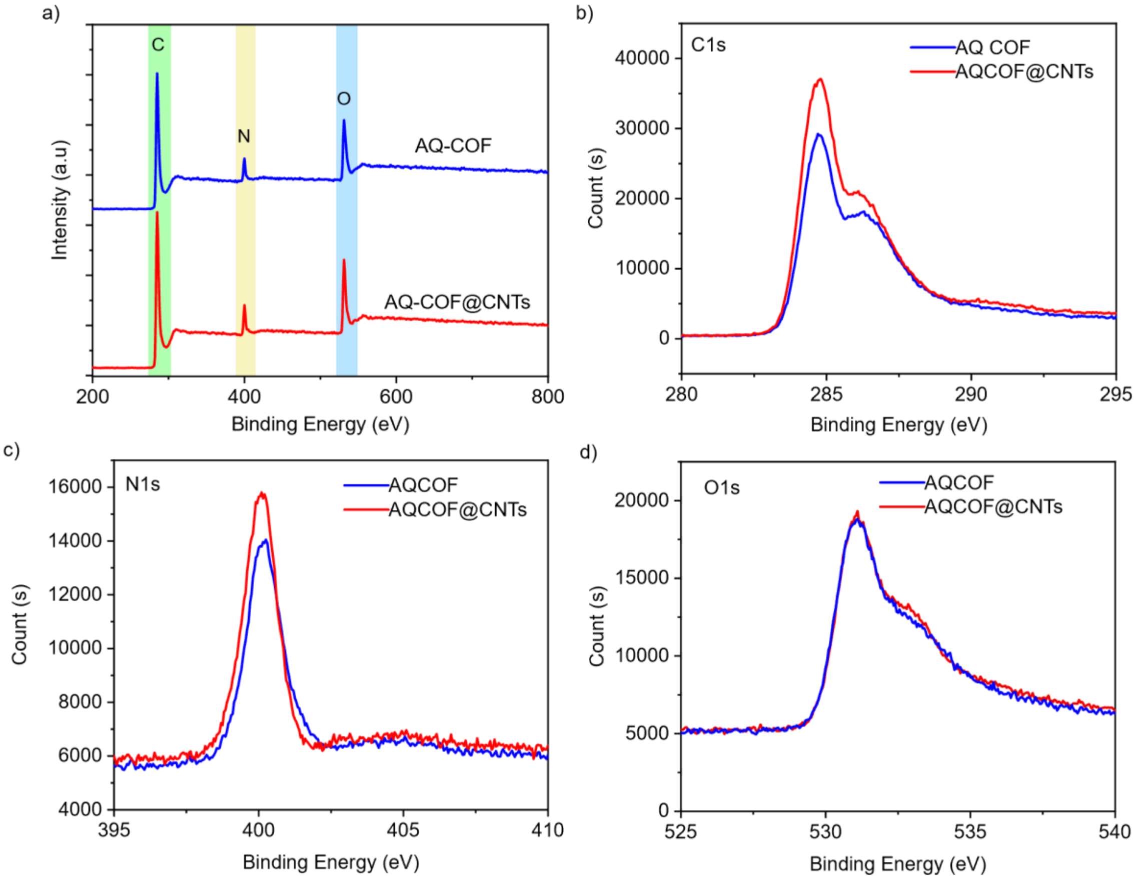

Figure S5. X-ray photoelectron spectroscopy (XPS) analysis of AQCOF and AQCOF@CNTs. (a) Survey XPS spectrum of AQCOF@CNTs, showing the overall elemental composition. High-resolution XPS spectra for (b) C 1s, (c) N 1s, and (d) O 1s, respectively. The binding energy peaks appear at similar positions for both AQCOF and AQCOF@CNTs, indicating that the chemical environments and bonding states of carbon, nitrogen, and oxygen remain largely unchanged upon incorporation of CNTs.

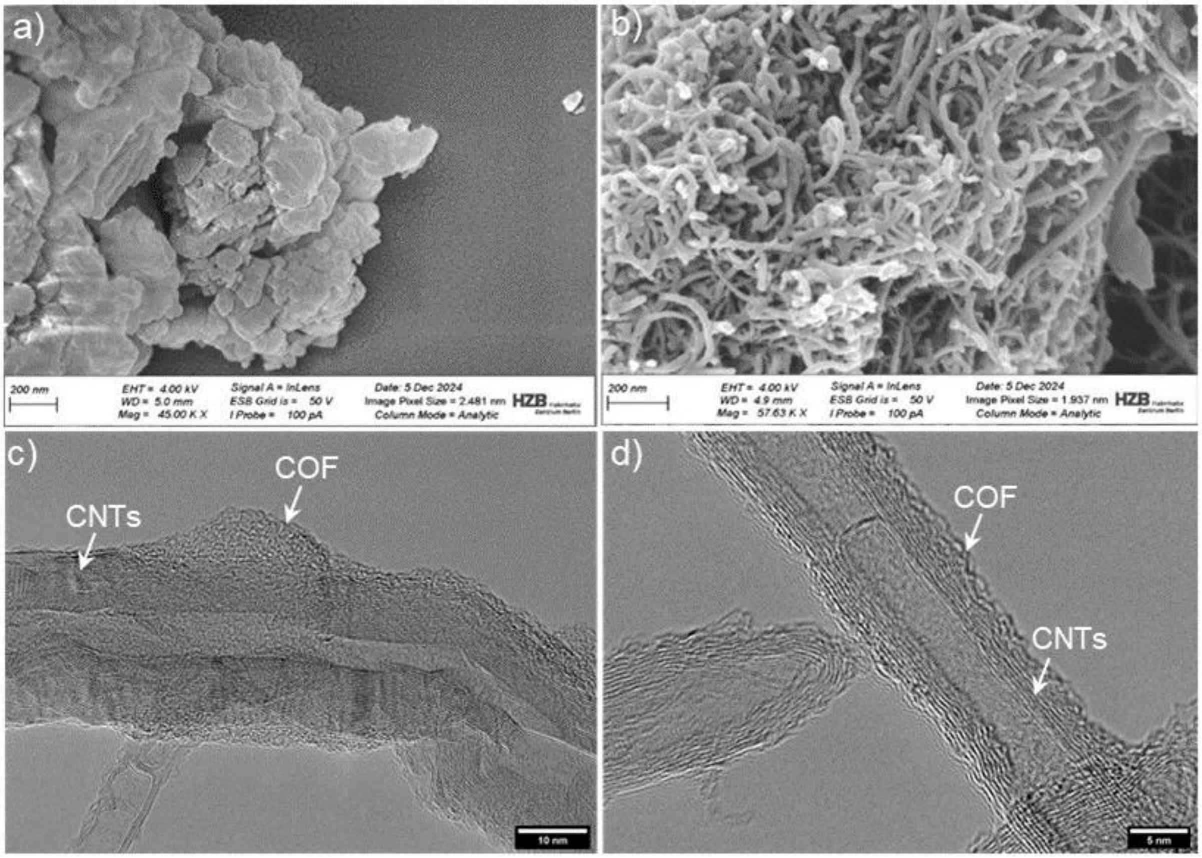

Figure S6. Electron microscopy analysis of pristine AQCOF and AQCOF@CNTs. Top: (a) SEM of pristine AQCOF and (b) AQCOF@CNTs. (Scale bar: 200 nm). (c-d) HRTEM images AQCOF@CNTs (Scale bar: 10 and 5 nm respectively), showing COF growth on CNTs forming well integrated structure.

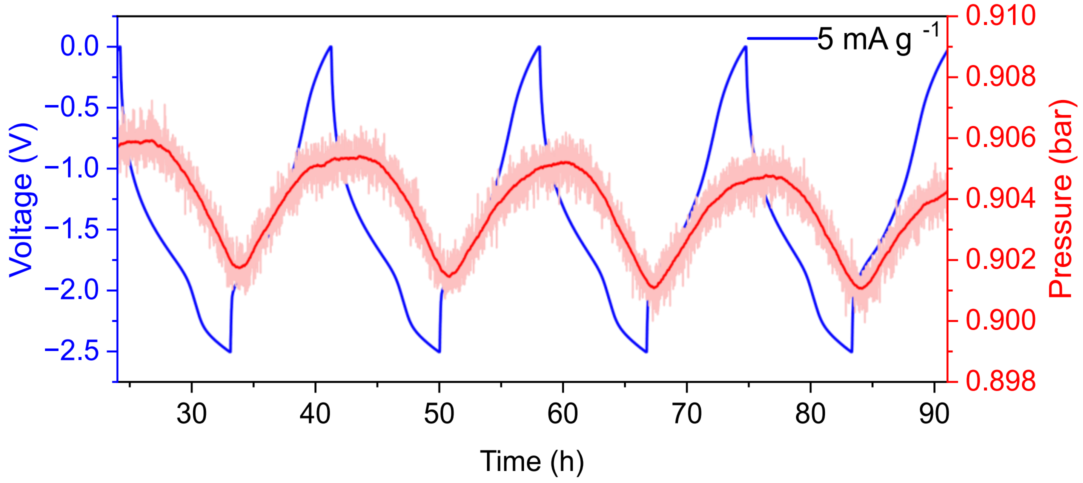

Figure S7. Electrochemical CO_2 uptake performance of the AQCOF-based device using an ionic liquid electrolyte. (a) Galvanostatic charge–discharge (GCD) profiles (blue) and corresponding CO_2 pressure changes (red) were recorded in a cell having configuration 'CO_2 /AQCOF@CNTs /[Bmim][TFSI] /YP80F' under a CO_2 atmosphere. Measurements were performed at current densities ranging from 5 mA g^{-1} in negative charging mode, with a 5-minute potential hold at the vertices each cycle. Measurements are conducted in static mode. Pressure data were smoothed using a moving average every 100 seconds.

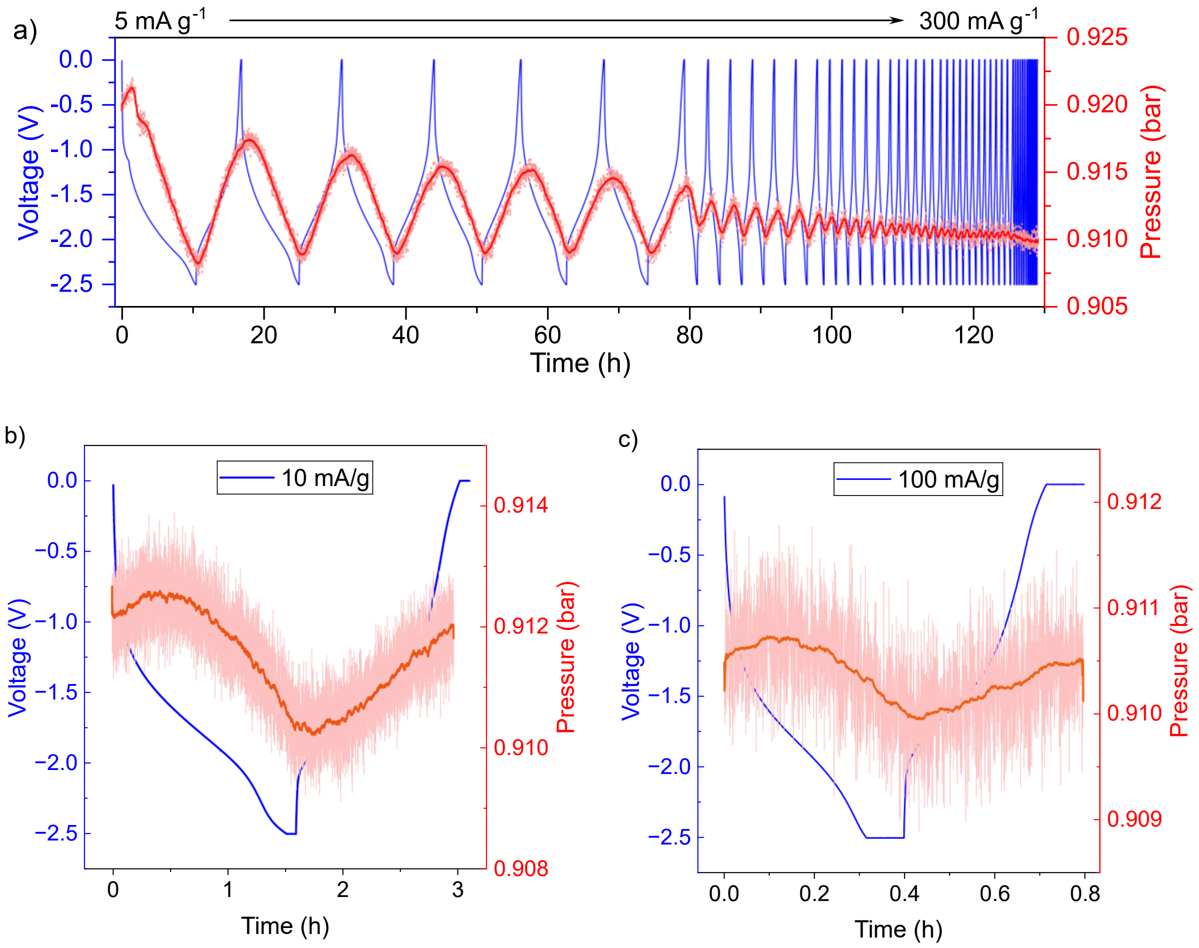

Figure S8. Electrochemical CO_2 uptake performance of the AQCOF@CNTs-based device using an ionic liquid electrolyte. (a) Galvanostatic charge–discharge (GCD) profiles (blue) and corresponding CO_2 pressure changes (red) were recorded in a cell having configuration 'CO_2 / AQCOF@CNTs / [Bmim][TFSI] / YP80F' under a CO_2 atmosphere. Measurements were performed at current densities ranging from 5 to 300 mA g^{-1} in negative charging mode, with a 5-minute potential hold at the vertices each cycle. The figures (b, and c) are enlarged GCD curves (blue) and corresponding pressure changes (red) recorded during CO_2 capture and release cycles at 10 mA g^{-1} and 100 mA g^{-1}, respectively. All the measurements are conducted in static mode. Pressure data were smoothed using a moving average every 100 seconds.

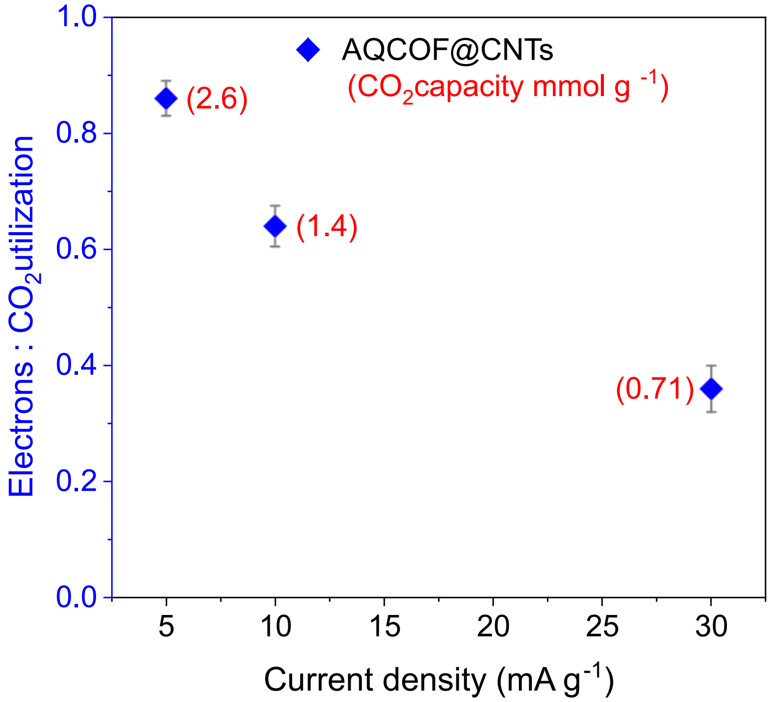

Figure S9. Electron utilization ratio of the AQCOF@CNTs electrode at varying current densities (ionic liquid cell). Measurements were conducted under a CO_2 atmosphere (~1 bar) using [Bmim][TFSI] (1-butyl-3-methylimidazolium bis(trifluoromethylsulfonyl)imide) as the organic electrolyte. The electron utilization was evaluated at current densities of 5, 10, and 30 mA g^{-1} to assess the efficiency of charge-to-adsorption conversion across different operating conditions.

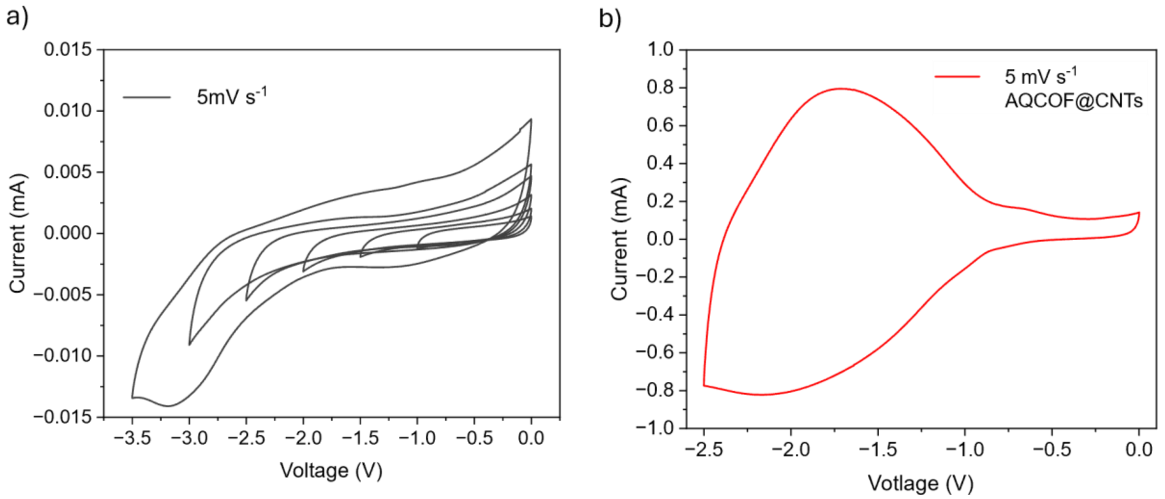

Figure S10. Stability window of the ionic liquid electrolyte determined in a blank cell. (a) Cyclic voltammetry of 1-butyl-3-methylimidazolium bis(trifluoromethylsulfonyl)imide electrolyte in electrochemical device without COF and carbon electrodes. The electrolyte remains stable within 0 to –2.5 V, only showed a small faradaic current in this range. (b) CV of the device having AQCOF@CNTs as working electrode, [Bmim][TFSI] as electrolyte, YP80F counter electrode. The measurement was performed at a scan rate of 5 mV s^{-1}.

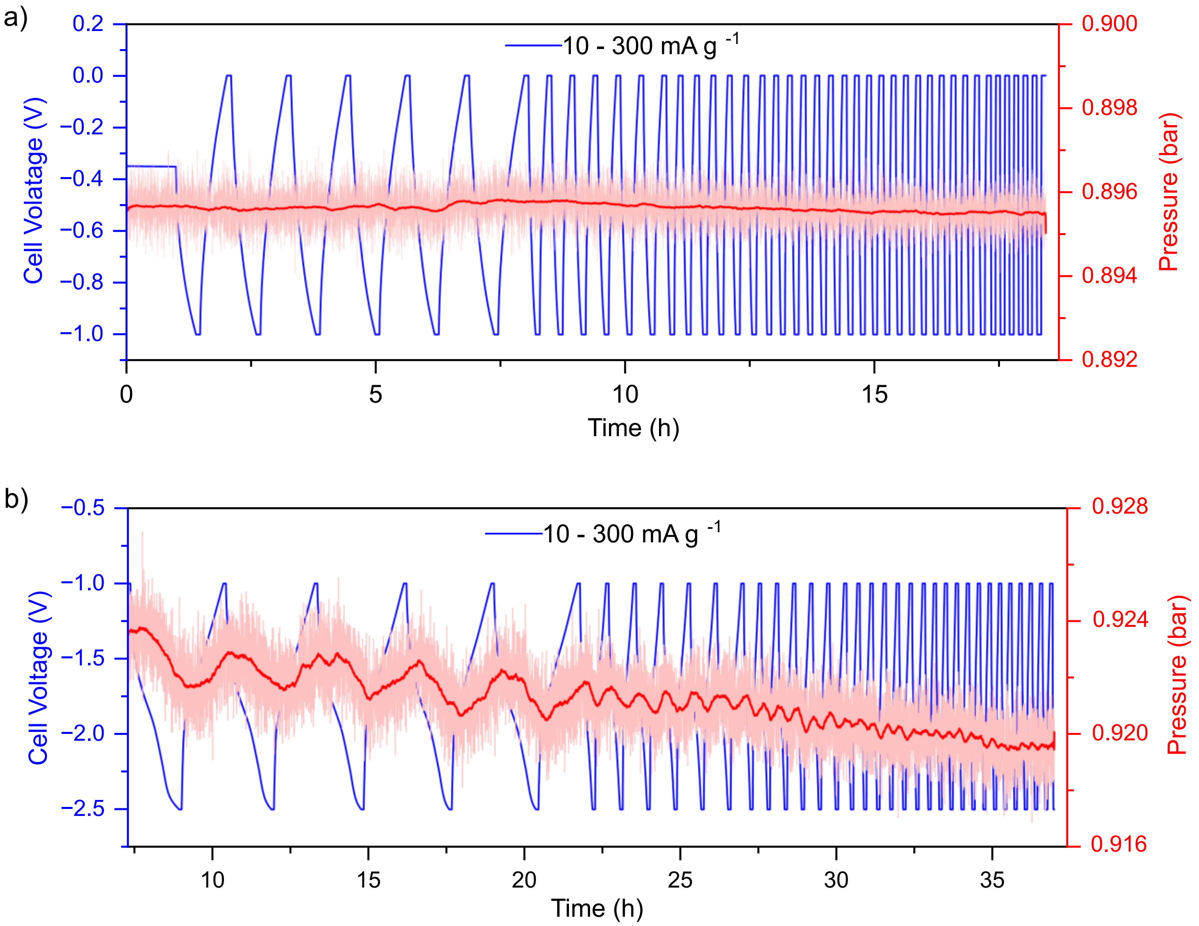

Figure S11. The effect of breaking the working voltage windows into different voltage regimes on electrochemical CO_2 capture capacity (ionic liquid cell). (a) Electrochemical CO_2 uptake measurement using CO_2 AQCOF@CNTs /[Bmim][TFSI] / YP80F under 100% CO_2 atmosphere operating between the voltage window 0 to -1 V at current densities and from 10, 30, 50 70, 90, 100, and 300 mA g^{-1}. (b) CO_2 uptake measurements conducted by extending voltage regime from -1.0 to -2.5 with all other experimental conditions unchanged. In both cases, the device was negatively charged, and a 5 min voltage hold was maintained between the charge-discharged cycles. All the measurements are conducted in static mode. Pressure data were smoothed using a moving average of every 100 seconds. Note: Quantified CO_2 uptake values for the respective regimes are given in the main text (Figure 2d).

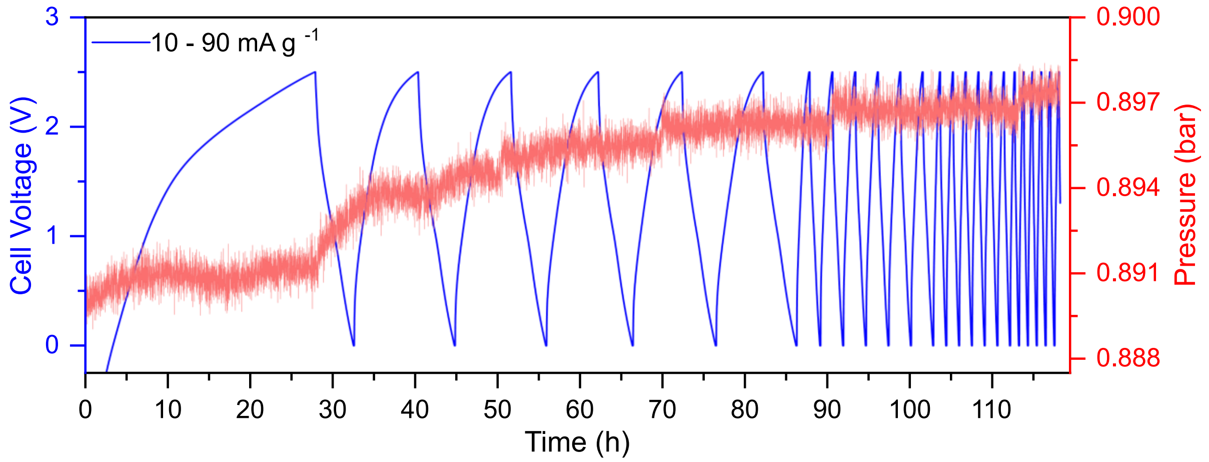

Figure S12. CO_2 uptake performance of the AQCOF@CNTs-based electrochemical cell under positive polarization (ionic liquid cell). (a) Galvanostatic charge–discharge (GCD) curves (blue) and corresponding pressure variations (red) recorded for CO_2 in a cell configured as '100% CO_2 / AQCOF@CNTs / [Bmim][TFSI] / YP80F'. The cell was operated at current densities of 10, 30, 50, and 90 mA g^{-1} within a voltage window of 0 to 2.5 V, operated in a static mode with a 5-minute voltage hold during cycles. Note: The observed pressure sharp increase in internal pressure of the cell during positive charging indicates potential device failure and the likely occurrence of parasitic reactions.

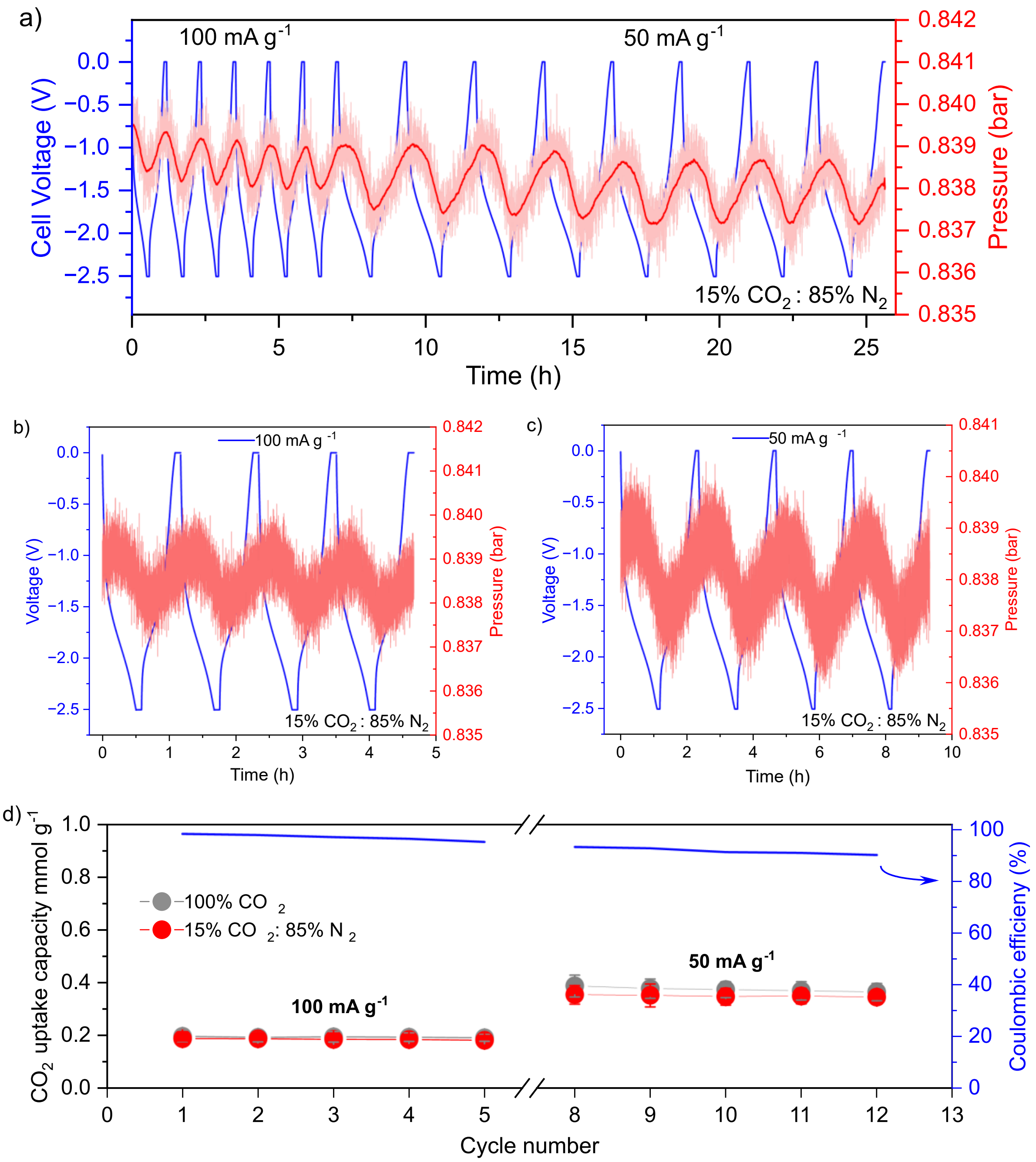

Figure S13. Evidence of CO_2 selectivity of AQCOF@CNTs based electrochemical device under various gas mixtures (ionic liquid cells). (a) Galvanostatic charge–discharge profiles (blue) and corresponding pressure changes (red) of gas mixture using electrochemical cell with the configuration: '15% CO_2–85% N_2/AQCOF@CNTs /[Bmim][TFSI] / YP80F', operated at current densities of 100 mA g^{-1} and 50 mA g^{-1} with a voltage range set between 0 to –2.5 V. (b, c) Enlarged views of GCD cycles highlighting CO_2 pressure variations in the 15% CO_2–85% N_2 mixture at 100 mA g^{-1} and 50 mA g^{-1}, respectively. (d) Quantified CO_2 uptake under 100 % CO_2 and mixed gas conditions, alongside the corresponding coulombic efficiency across cycles under 100% CO_2 and gas mixtures containing 15% CO_2.

Note: The pressure response (uptake/release) in gas mixtures follows a similar trend to that observed in pure CO_2, supporting selective CO_2 interaction. All experiments were conducted under negative polarization with a 5-minute voltage hold. All the measurements are conducted in static mode. Pressure data were smoothed using a moving average every 100 seconds. Error bars represent the 95% confidence interval calculated using Student's t-test.

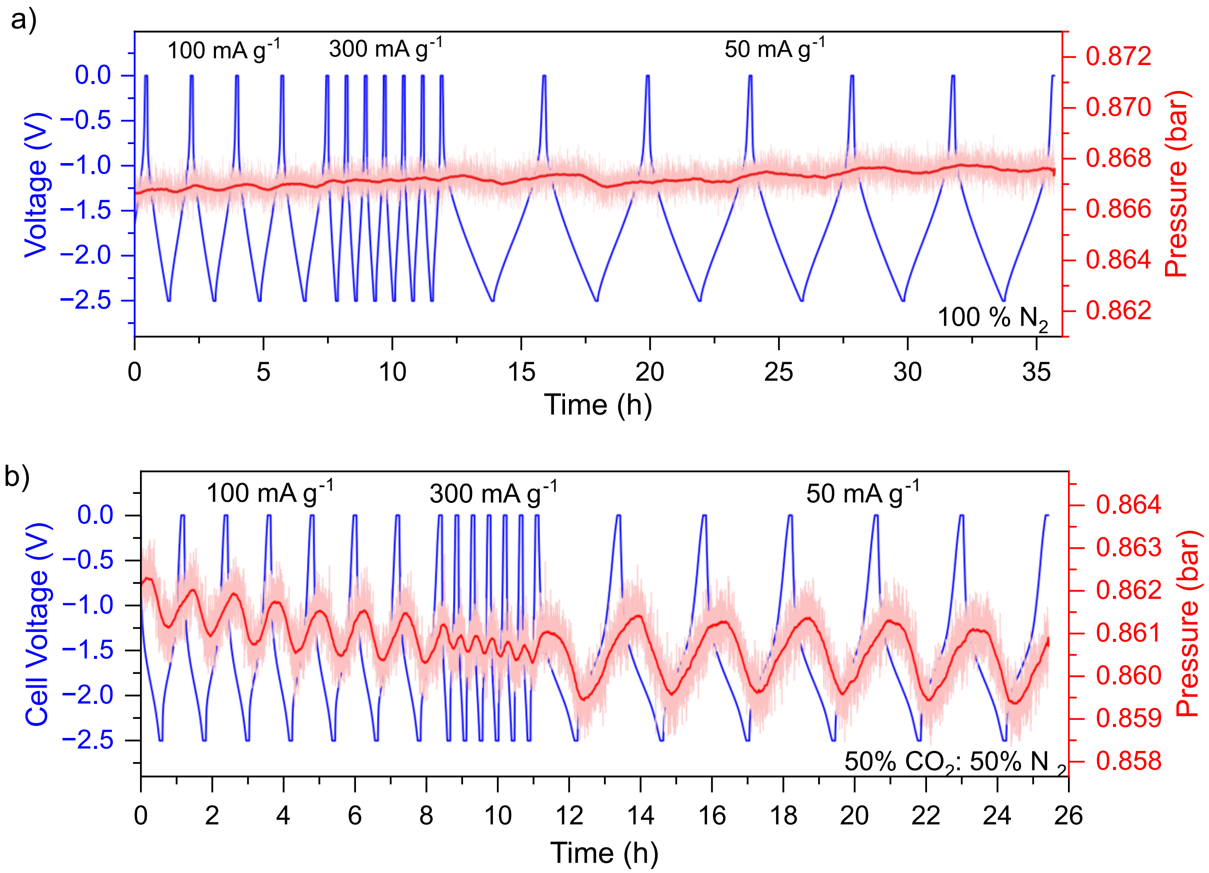

Figure 14. Evidence of CO_2 selectivity of AQCOF@CNTs based electrochemical device in different gas mixtures (ionic liquid cells). (a) Galvanostatic charge–discharge (GCD) profiles (blue) and corresponding pressure variations (red) recorded under a 100% N_2 atmosphere using an electrochemical cell with the configuration: '100% N_2 /AQCOF@CNTs /[Bmim][TFSI] / YP80F'. Experiments were conducted at current densities of 50, 100, and 300 mA g^{-1} within a voltage range of 0 to –2.5 V. (b) Pressure changes under a 50% N_2 : 50% CO_2 gas mixture using the same device configuration at different current densities (100, 300, and 50 mA g^{-1}). All measurements were conducted under static gas conditions and negative polarization, with a 5-minute voltage hold per cycle. Pressure data were smoothed using a moving average every 100 seconds.

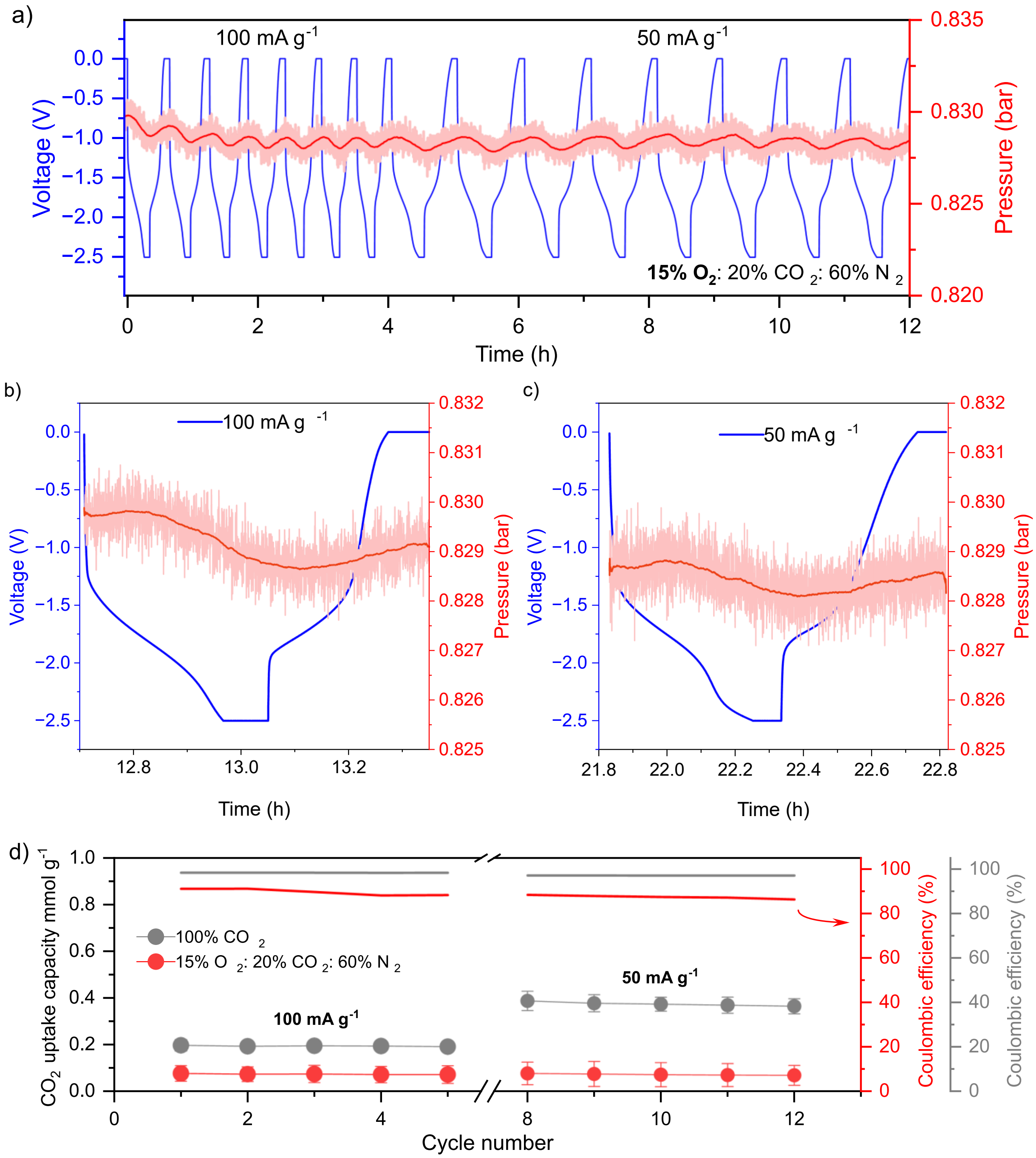

Figure S15. Evaluation of CO_2 selectivity of AQCOF@CNTs based electrochemical device under mixture containing oxygen (ionic liquid cells). (a) Galvanostatic charge–discharge profiles (blue) and corresponding pressure changes (red) of gas mixture using electrochemical cell with the configuration: '15% O_2 / 20% CO_2 / 60% N_2 /AQCOF@CNTs /[Bmim][TFSI] / YP80F', operated at current densities of 100 mA g^{-1} and 50 mA g^{-1} with a voltage range set between 0 to –2.5 V. (b, c) Enlarged views of GCD cycles highlighting gas pressure variations in the 15% O_2/ 20% CO_2 / 60% N_2 mixture at 100 mA g^{-1} and 50 mA g^{-1}, respectively. (d) Quantified CO_2 uptake under 100 % CO_2 and mixed gas conditions (15% O_2:

20% CO_2: 60% N_2), alongside the corresponding coulombic efficiency under 100% CO_2 and gas mixtures containing 15% O_2.

Note: The pressure response (uptake/release) in gas mixtures follows a similar trend to that observed in pure CO_2, supporting selective CO_2 interaction. All experiments were conducted under negative polarization with a 5-minute voltage hold. All the measurements are conducted in static mode. In gas mixtures containing N_2, CO_2 and O_2 we assumed the pressure changes come solely from CO2 uptake/release, rather than N_2 uptake/release. In 100% N2 quantified pressure change values fell within the measurement uncertainty. We Pressure data were smoothed using a moving average every 100 seconds. Error bars represent the 95% confidence interval calculated using Student's t-test.

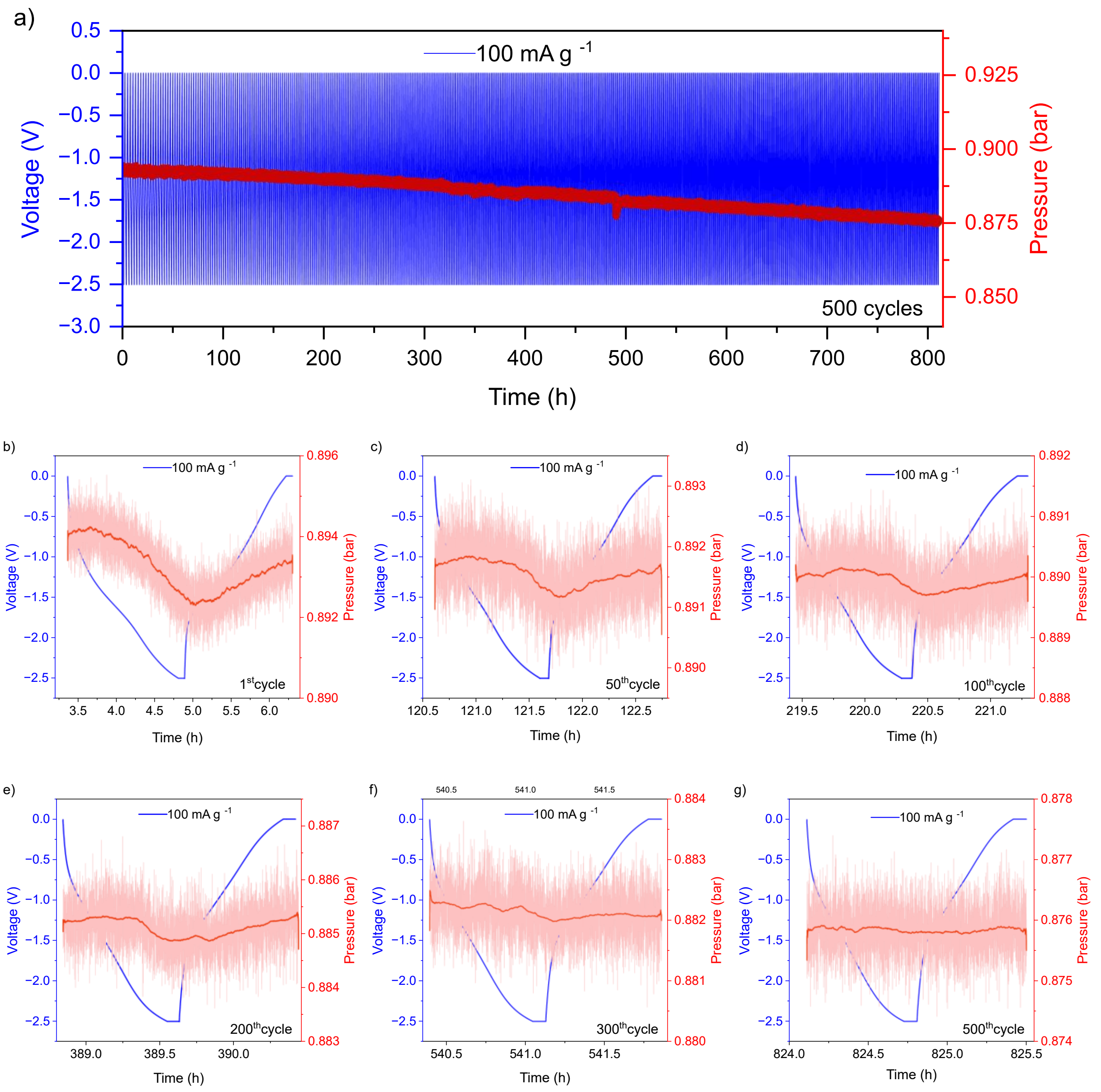

Figure S16. Long-cycling CO_2 capture performance of the AQCOF@CNTs-based electrochemical device (ionic liquid cell). (a) The device having configuration 'CO_2 / YP80F /[Bmim][TFSI]/ YP80F', was operated within a voltage window of 0 to –2.5 V under a 100% CO_2 atmosphere. Galvanostatic charge–discharge profiles (blue) and corresponding pressure variations (red) were recorded at a current density of 100 mA g^{-1}, with 5-minute voltage holds during each cycle. CO_2 adsorption capacities were normalized to the mass of the active material in the working electrode. A gradual capacity fade was noticed; however, the system demonstrated a high average Coulombic efficiency of ~91.6%, suggesting enhanced electrochemical stability. Importantly, a ~20% capacity loss after 50 and 60 % loss was recorded after 100 cycles. After 300 cycles only 20% of the initial uptake capacity was retained. The figures (b-g) show enlarged 1st, 50th, 100th, 300th, and 500th cycle. All measurements were conducted under static gas conditions and negative polarization, with a 5-minute voltage hold per cycle. Pressure data were smoothed using a moving average every 100 seconds.

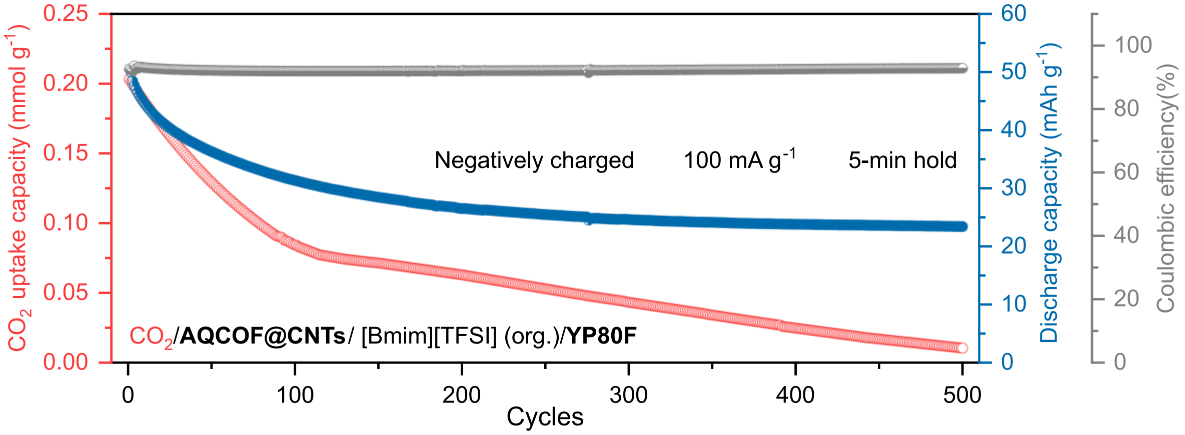

Figure S17. Long cycling performance of the AQCOF@CNTs-based electrochemical device (ionic liquid cell). (a) The device having configuration 'CO_2 / YP80F /[Bmim][TFSI]/ YP80F', was operated within a voltage window of 0 to –2.5 V under a 100% CO_2 atmosphere. The figure also shows discharge capacity, CO_2 uptake capacities and Coulombic efficiencies at the current density of 100 mA g^{-1} in the negative charging mode, with 5-min voltage hold.

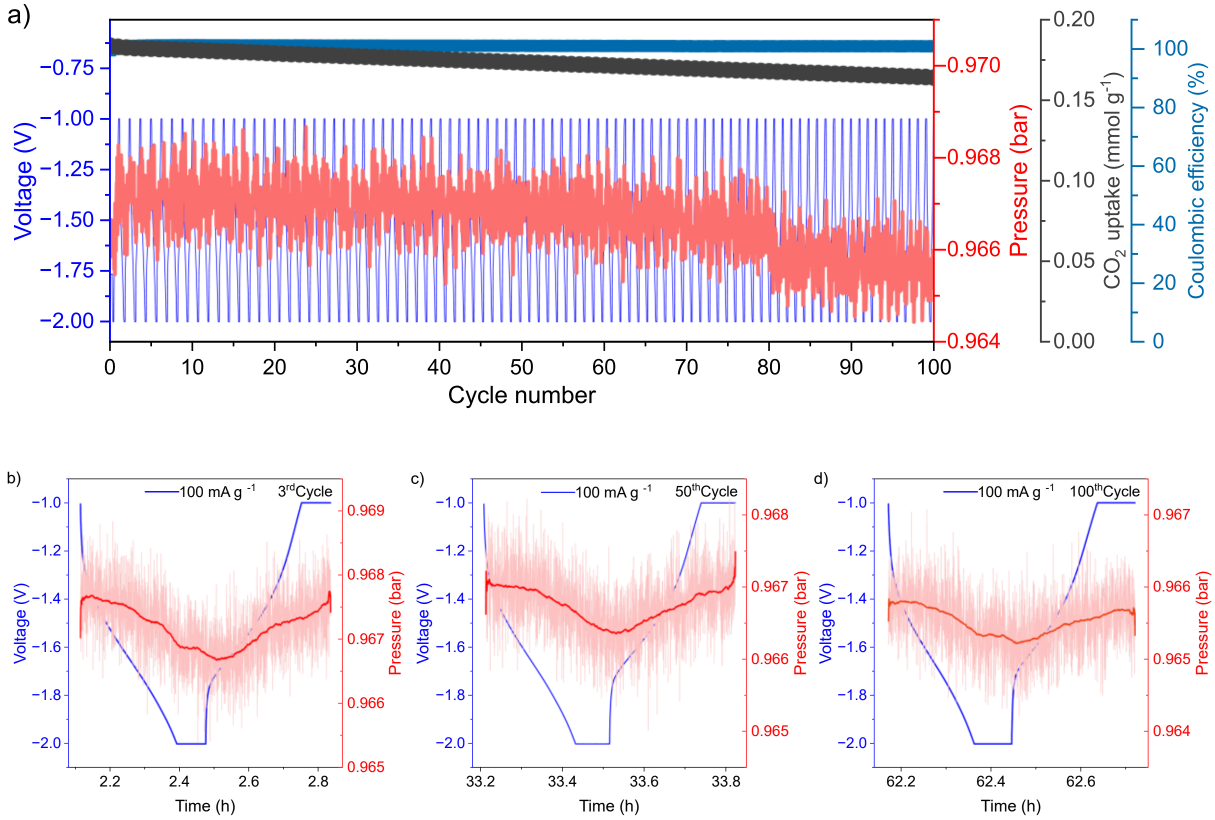

Figure S18. Long-term CO_2 capture performance of the AQCOF@CNTs-based electrochemical device (ionic liquid cell). (a) The device having configuration 'CO_2 / YP80F /[Bmim][TFSI]/ YP80F', was operated within a voltage window of 0 to –2.0 V under a 100% CO_2 atmosphere. Galvanostatic charge–discharge profiles (blue) and corresponding pressure variations (red) were recorded at a current density of 100 mA g^{-1}, with 5-minute voltage holds during each cycle. CO_2 adsorption capacities were normalized to the mass of the active material in the working electrode. Although the overall CO_2 uptake capacity was relatively low, the system demonstrated a high average Coulombic efficiency of ~98.3%, suggesting enhanced electrochemical stability compared to the broader voltage range operation (0 to –2.5 V, Figure S15), likely due to the suppression of parasitic side reactions. Importantly, only a ~10.7% reduction in CO_2 uptake capacity was observed over 100 cycles, in stark contrast to the ~60% capacity loss observed under extended voltage conditions, indicating significantly improved long-term cycling stability at the optimized voltage range. The figure (b) is the enlarged 3rd cycle, (c) is 50th cycle and (d) is the 100th cycle. All measurements were conducted under static gas conditions and negative polarization, with a 5-minute voltage hold per cycle. Pressure data were smoothed using a moving average every 100 seconds.

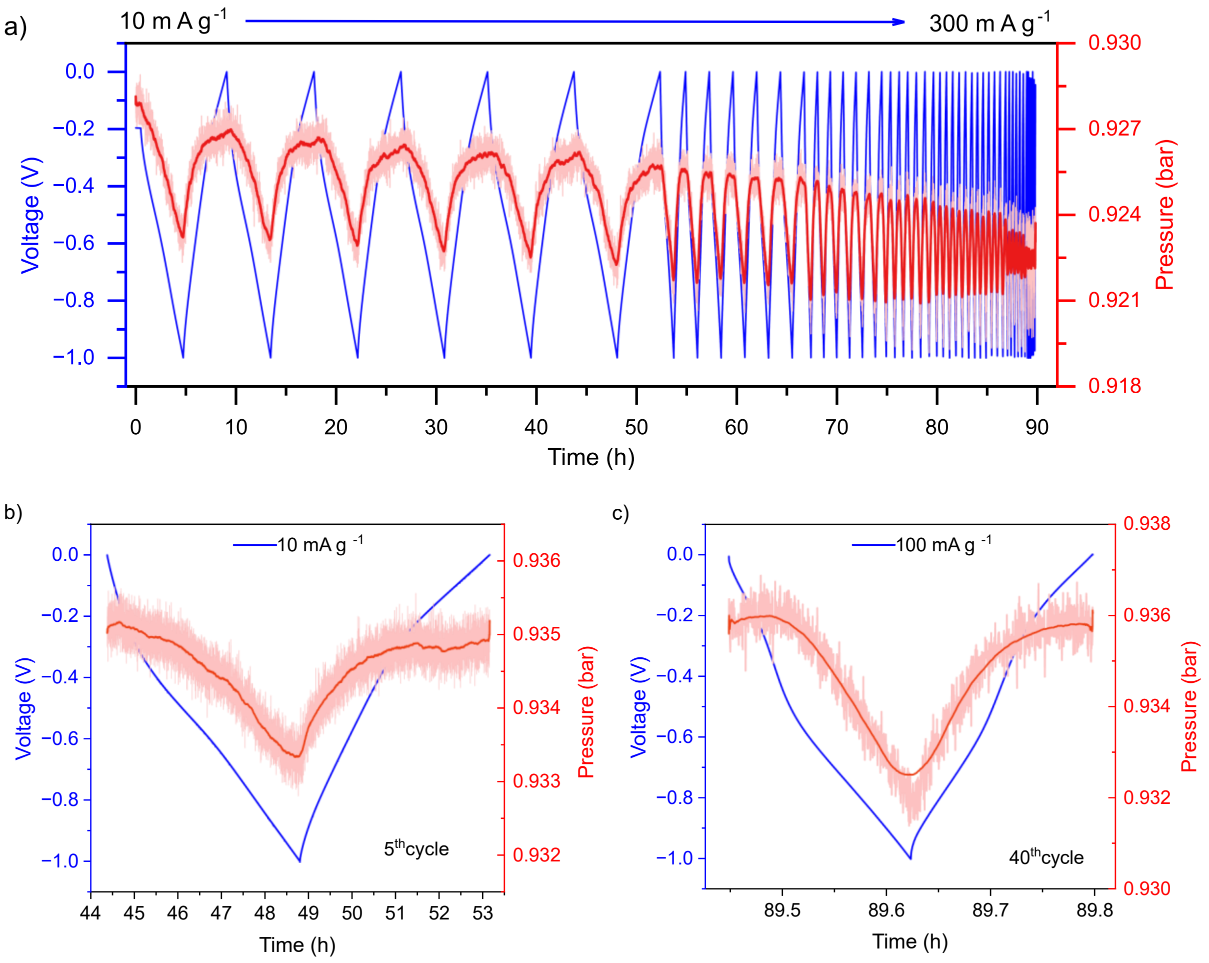

Figure S19. Electrochemical CO_2 uptake measurements of AQCOF@CNTs material in 1 M Na_2SO_4 electrolyte system under CO_2 atmosphere (~1 bar). (a) Galvanostatic charge-discharge profiles (bule) and corresponding CO_2 pressure changes (red) in device with a cell configuration of 'CO_2/AQCOF@CNTs/ 1 M Na_2SO_4 (aq. electrolyte)/ YP80F' at different current densities from 10 mA g^{-1} to 300 mA g^{-1}. All the measurements are conducted in negative charging mode without voltage hold. The enlarged GCD data (blue) and pressure change smoothed moving averaged every 100 seconds (red) of a CO_2 capture(release) cycle measured at (b) 10 mA g^{-1} and (c) 100 mA g^{-1} respectively. All measurements were conducted under static gas conditions and negative polarization, with a 5-minute voltage hold per cycle. Pressure data were smoothed using a moving average every 100 seconds.

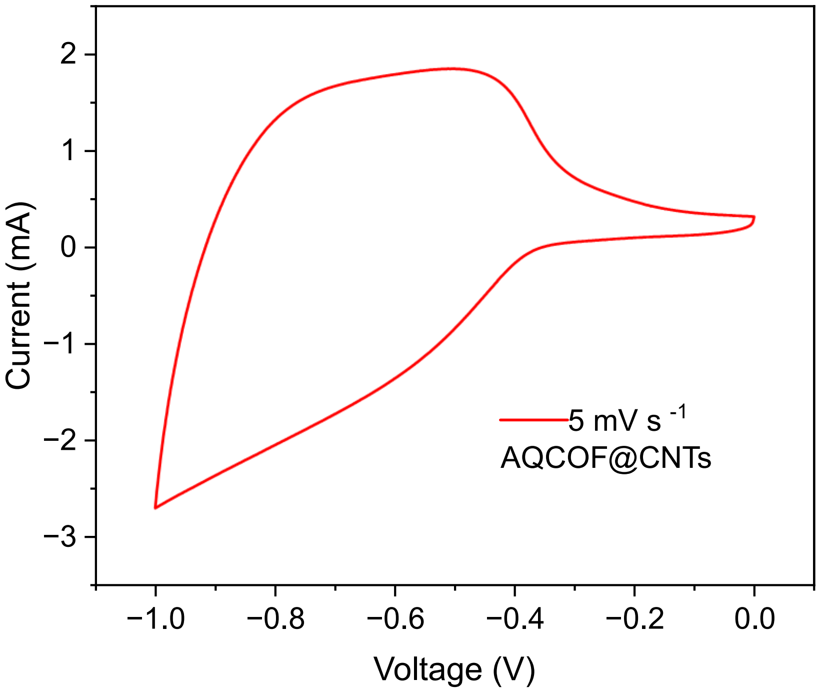

Figure S20. Cyclic voltammetry (CV) profile of the AQCOF@CNTs-based electrochemical device employing an aqueous electrolyte. The device configuration consists of CO_2/AQCOF@CNTs / 1 M Na_2SO_4 / YP80F, where 1 M Na_2SO_4 serves as the aqueous electrolyte. The measurement was performed at a scan rate of 5 mV s^{-1} under CO_2.

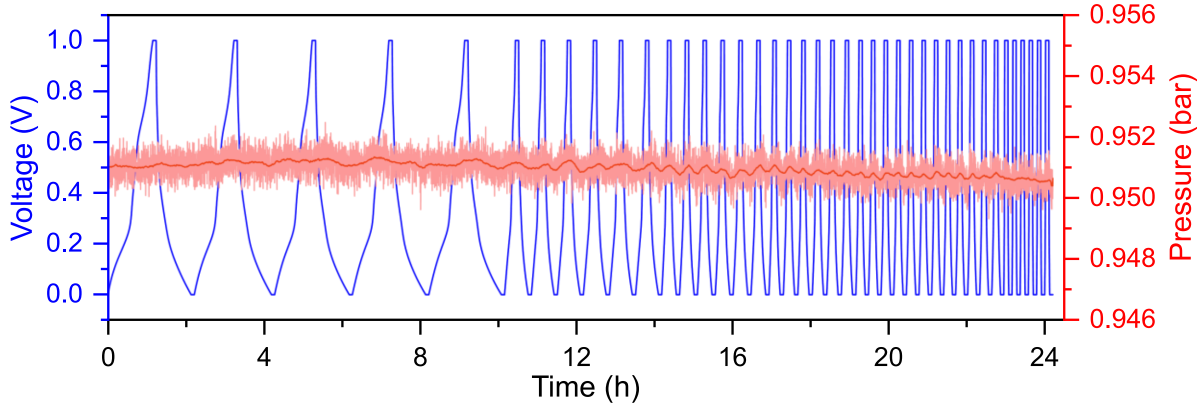

Figure S21. CO_2 uptake performance of the AQCOF@CNTs-based electrochemical cell under positive polarization (aqueous conditions). (a) Galvanostatic charge–discharge (GCD) curves (blue) and corresponding pressure variations (red) recorded for CO_2 in a cell configured as '100% CO_2 /AQCOF@CNTs / 1 M Na_2SO_4 /YP80F'. The cell was operated at current densities of 10, 30, 50, 70, 90, 100, and 300 mA g^{-1} within a voltage window of 0 to 1 V, in static mode with a 5-minute voltage hold per cycle. Measurements were conducted under static gas conditions and positive polarization, with a 5-minute voltage hold per cycle. Pressure data were smoothed using a moving average every 100 seconds.

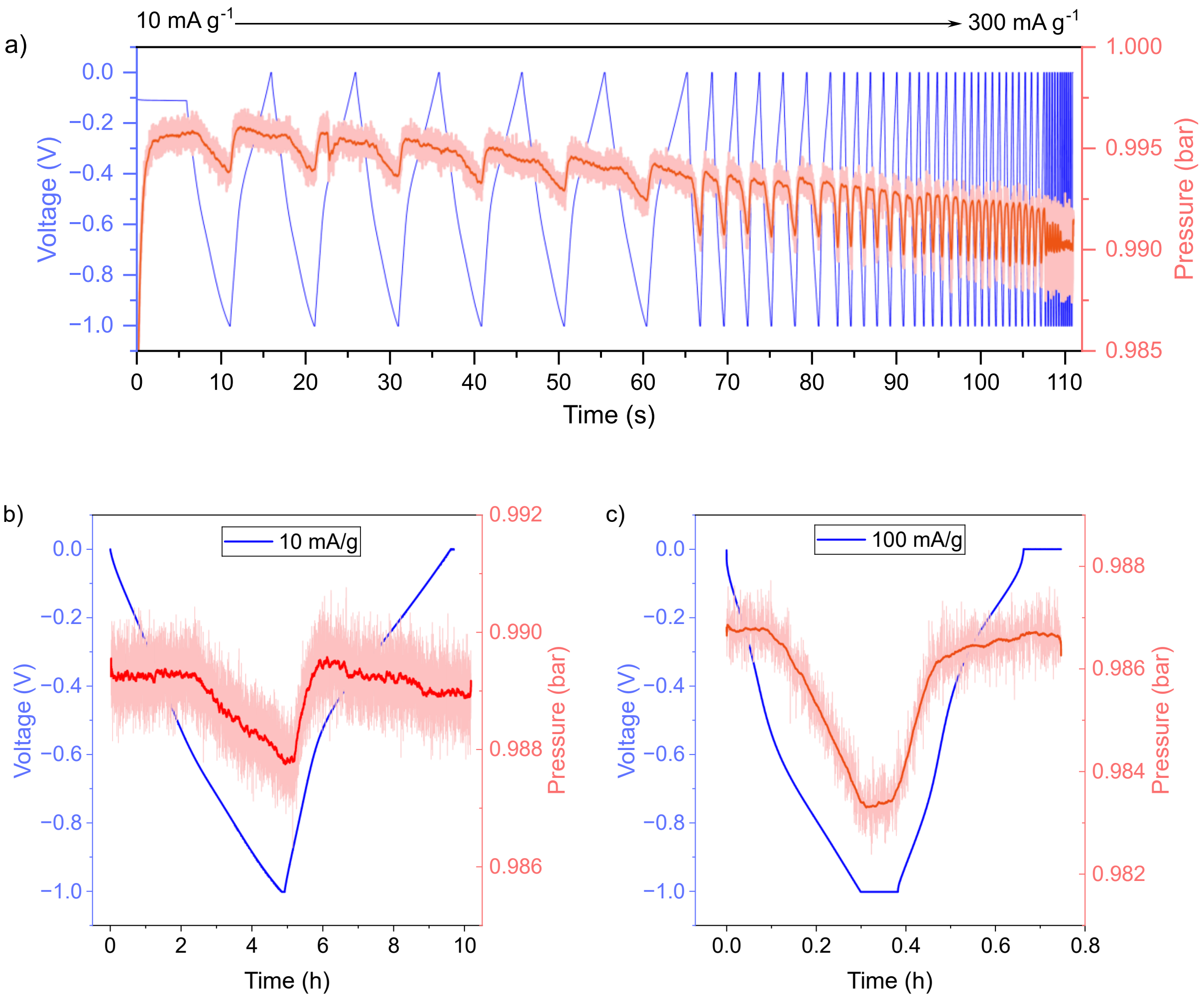

Figure S22. Electrochemical CO_2 uptake measurements of AQCOF@CNTs material in 1 M Na_2SO_4 electrolyte system under CO_2 atmosphere (~1 bar). (a) Galvanostatic charge-discharge profiles (bule) and corresponding CO_2 pressure changes (red) in device with a cell configuration of 'CO_2/AQCOF@CNTs/ 1 M Na_2SO_4 (aq. electrolyte)/ YP80F' at different current densities from 10 mA g^{-1} to 300 mA g^{-1}. All the measurements are conducted in negative charging mode with a 5-min voltage/potential hold. The enlarged GCD data (blue) and pressure change smoothed moving averaged every 100 seconds (red) of a CO_2 capture(release) cycle measured at (b) 10 mA g^{-1} and (c) 100 mA g^{-1} respectively. All measurements were conducted under static gas conditions and negative polarization, with a 5-minute voltage hold per cycle. Pressure data were smoothed using a moving average every 100 seconds.

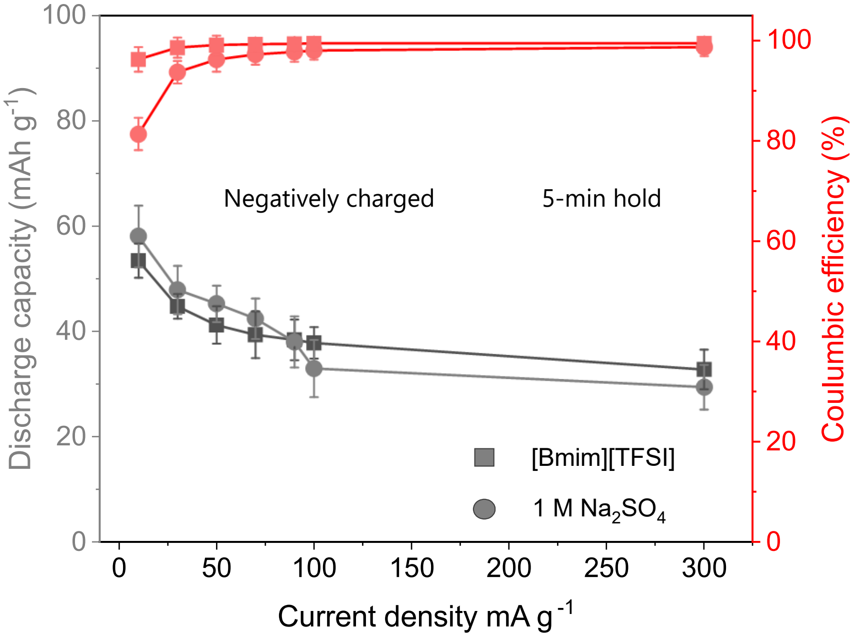

Figure S23. Comparison of the energy storage properties of AQCOF@CNTs-based electrochemical devices in ionic liquid and aqueous electrolytes. The electrochemical cells were configured as: (square) 100% CO_2 / AQCOF@CNTs / [Bmim][TFSI] / YP80F, and (sphere) 100% CO_2 / AQCOF@CNTs / 1 M Na_2SO_4 / YP80F. All experiments were conducted under static gas conditions and negative polarization, with a 5-minute voltage hold implemented in each cycle.

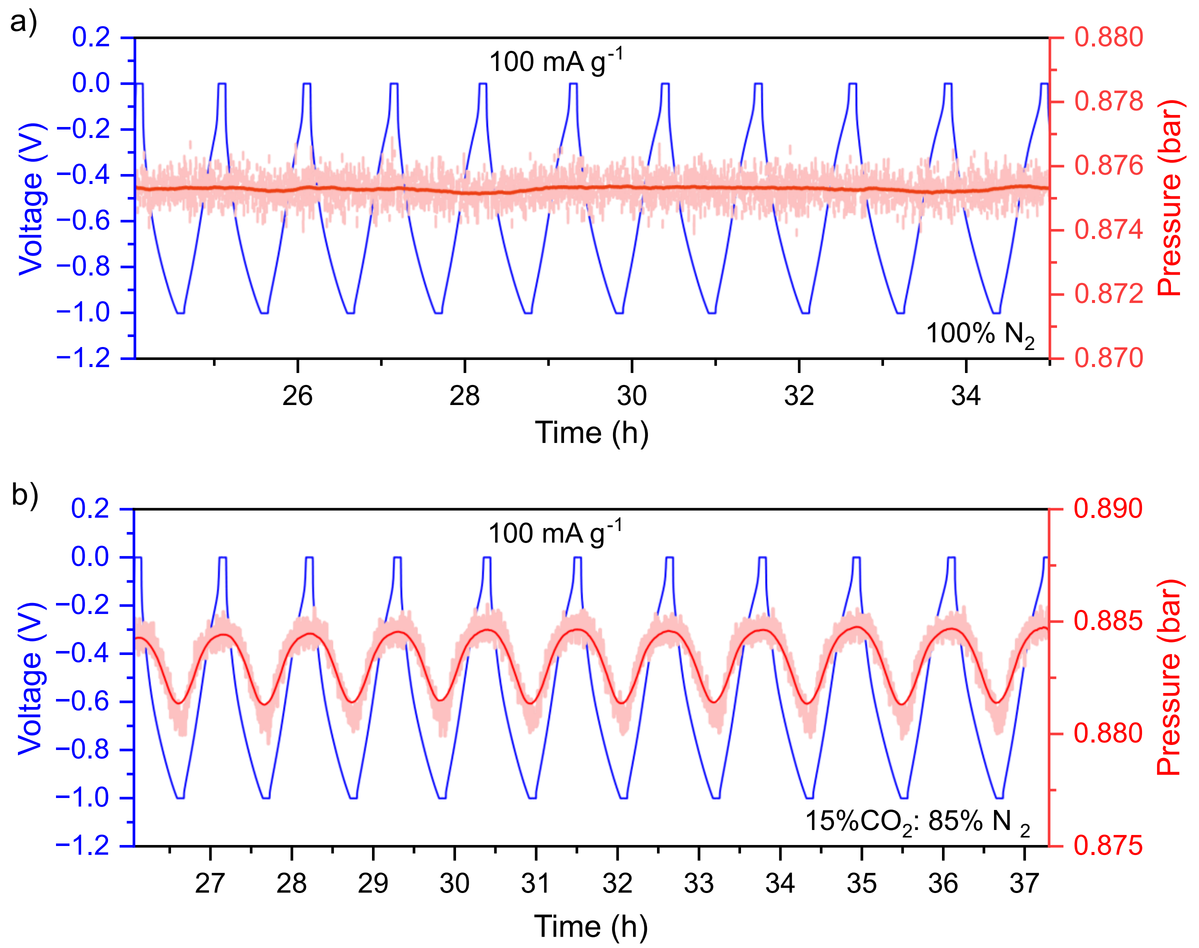

Figure S24. Experimental evidence of CO_2 selectivity in the AQCOF@CNTs-based electrochemical device in various gas mixtures under aqueous conditions. (a) Galvanostatic charge–discharge (GCD) curves (blue) and corresponding pressure variations (red) recorded under a 100% N_2 atmosphere using an electrochemical cell with the configuration: '100% N_2 /AQCOF@CNTs / 1 M Na_2SO_4 / YP80F'. Measurements were performed at a current density of 100 mA g^{-1} within a voltage range of 0 to –1 V. (b) Pressure response under an 85% N_2 : 15% CO_2 gas mixture using the same device configuration and current density. All experiments were conducted under static gas conditions and negative polarization, with a 5-minute voltage hold implemented in each cycle. Pressure data in b and c were smoothed using every 100-second moving average.

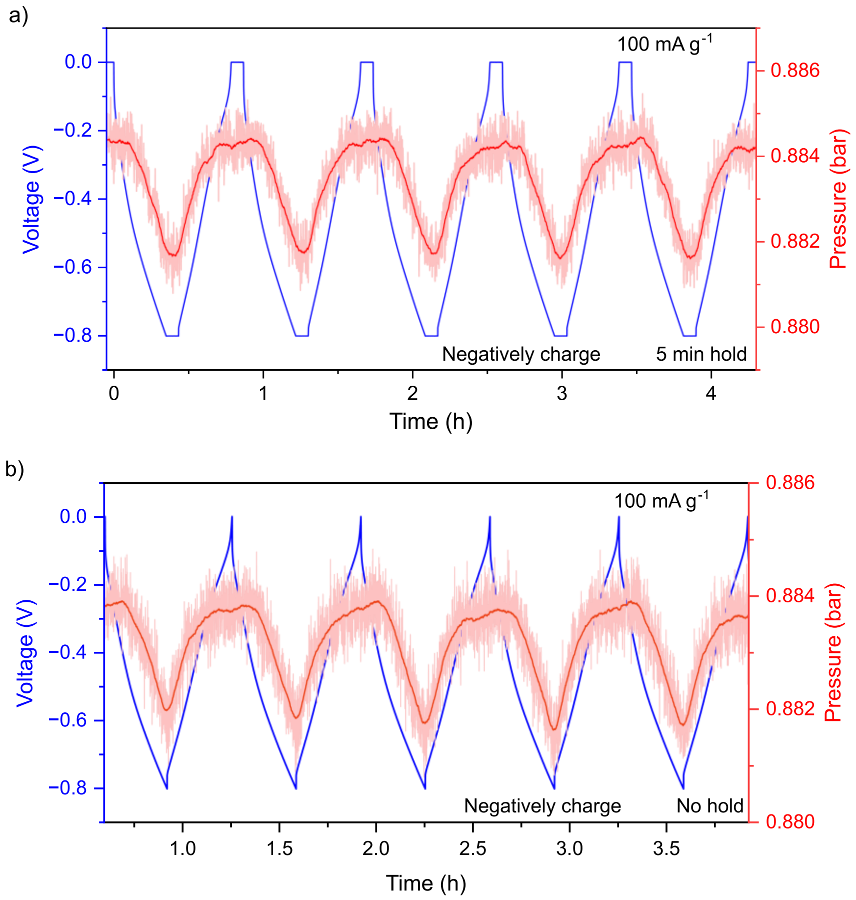

Figure S25. Optimization of the AQCOF@CNTs-based electrochemical device for CO_2 capture under aqueous conditions. (a) Galvanostatic charge–discharge (GCD) profiles (blue) and corresponding pressure variations (red) recorded under a 100% CO_2 atmosphere. Measurements were conducted under static gas conditions and negative polarization, with a 5-minute voltage hold applied during each cycle. The current density was set to 100 mA g^{-1}, and the voltage range was 0 to –0.8 V. (b) Pressure response under identical conditions but without a voltage hold, using the same device configuration and current density. Pressure data were smoothed using every 100-second moving average.

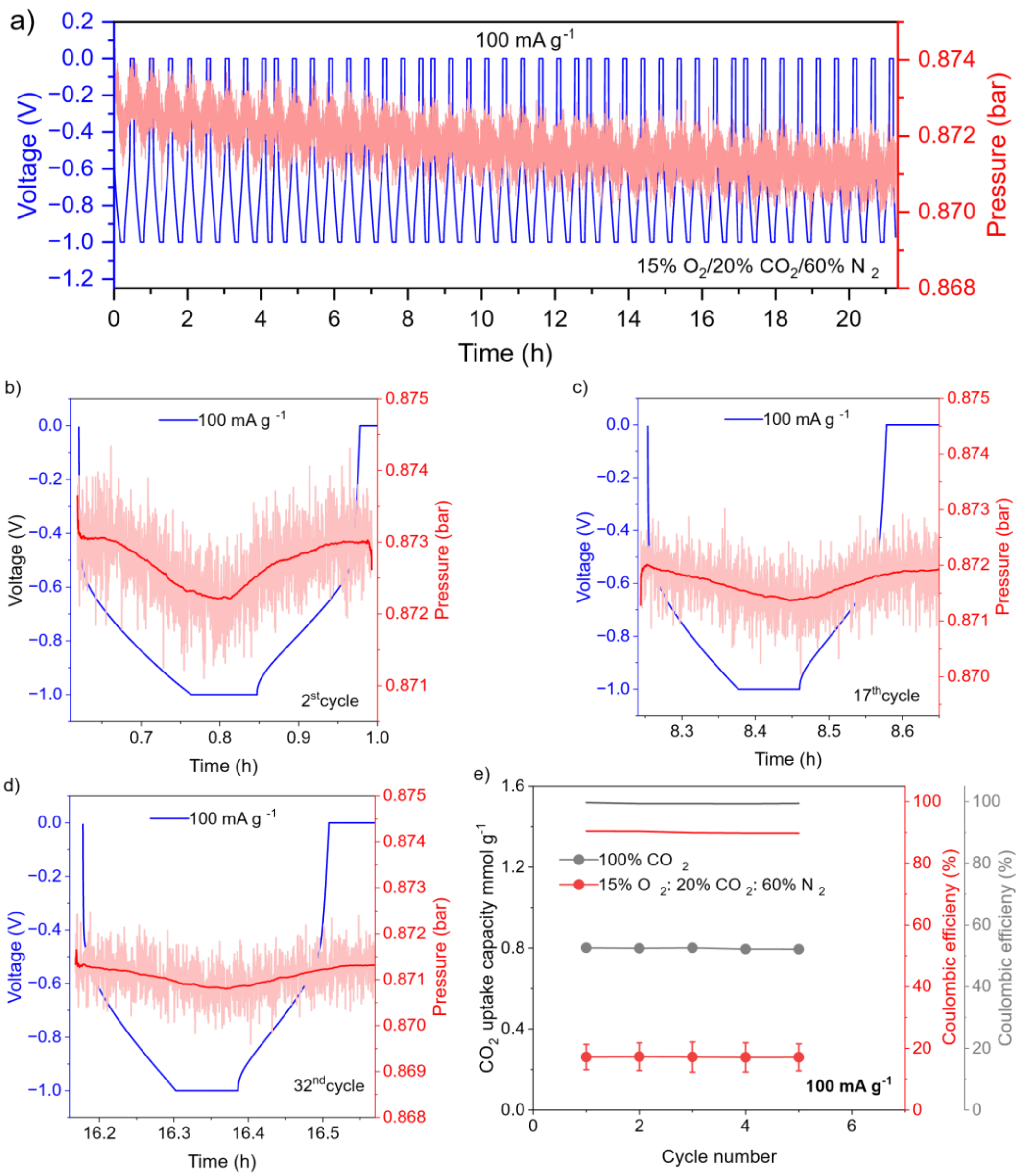

Figure S26. Selectivity evaluation of AQCOF@CNTs-based electrochemical device in gas mixtures containing oxygen under aqueous conditions. (a) Galvanostatic charge–discharge (GCD) curves (blue) and corresponding pressure variations (red) recorded under a 15% O_2: 20% CO_2: 60% N_2 atmosphere using an electrochemical cell with the configuration: '15% O_2: 20% CO_2: 60% N_2 /AQCOF@CNTs / 1 M Na_2SO_4 / YP80F'. Measurements were performed at a current density of 100 mA g^{-1} within a voltage range of 0 to –1 V. (b-d) Pressure change in 1st, 20th and 40th cycle. (e) Quantified CO_2 uptake under 100 % CO_2 and mixed gas conditions (15% O_2: 20% CO_2: 60% N_2), alongside the corresponding coulombic efficiencies across cycles under 100% CO_2 and gas mixtures containing 15% O_2. Note: All experiments were conducted under negative polarization with a 5-minute voltage hold. All the measurements are conducted in static mode. In gas mixtures containing N_2, CO_2 and O_2 we assumed the pressure changes come solely from CO_2 uptake/release, rather than N_2 uptake/release. In 100% N_2 quantified pressure change values fell within the measurement uncertainty. We Pressure data were smoothed using a moving average every 100 seconds. Error bars represent the 95% confidence interval calculated using Student's t-test

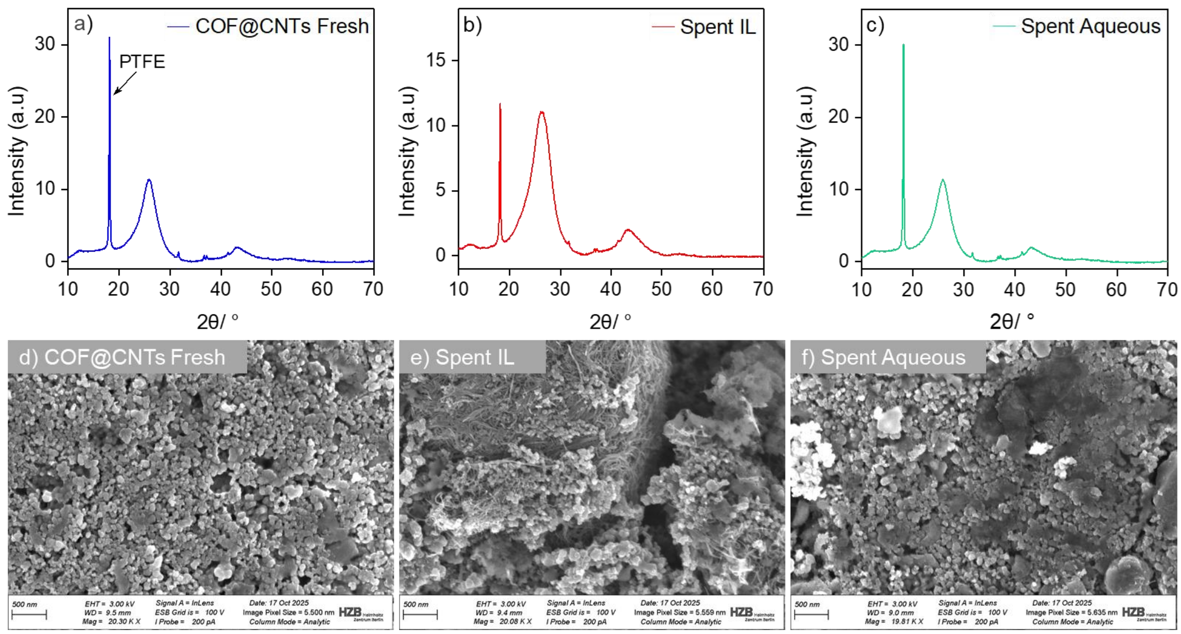

Figure 27. Structural stability of COF@CNT film electrodes after cycling in aqueous and ionic-liquid electrolytes. XRD (a-c) and SEM (d-f) analyses of COF@CNT electrode films before and after cycling. The COF@CNTs films show changes after long cycling. In ionic-liquid electrolytes, reduced PTFE peak intensity and visible cracks indicate film damage under high reductive currents, whereas in aqueous electrolyte they remain largely intact. Note: No change in electrolyte color was observed when electrochemical cells were opened for post cycling analysis. No gases evolved during operation of the device.

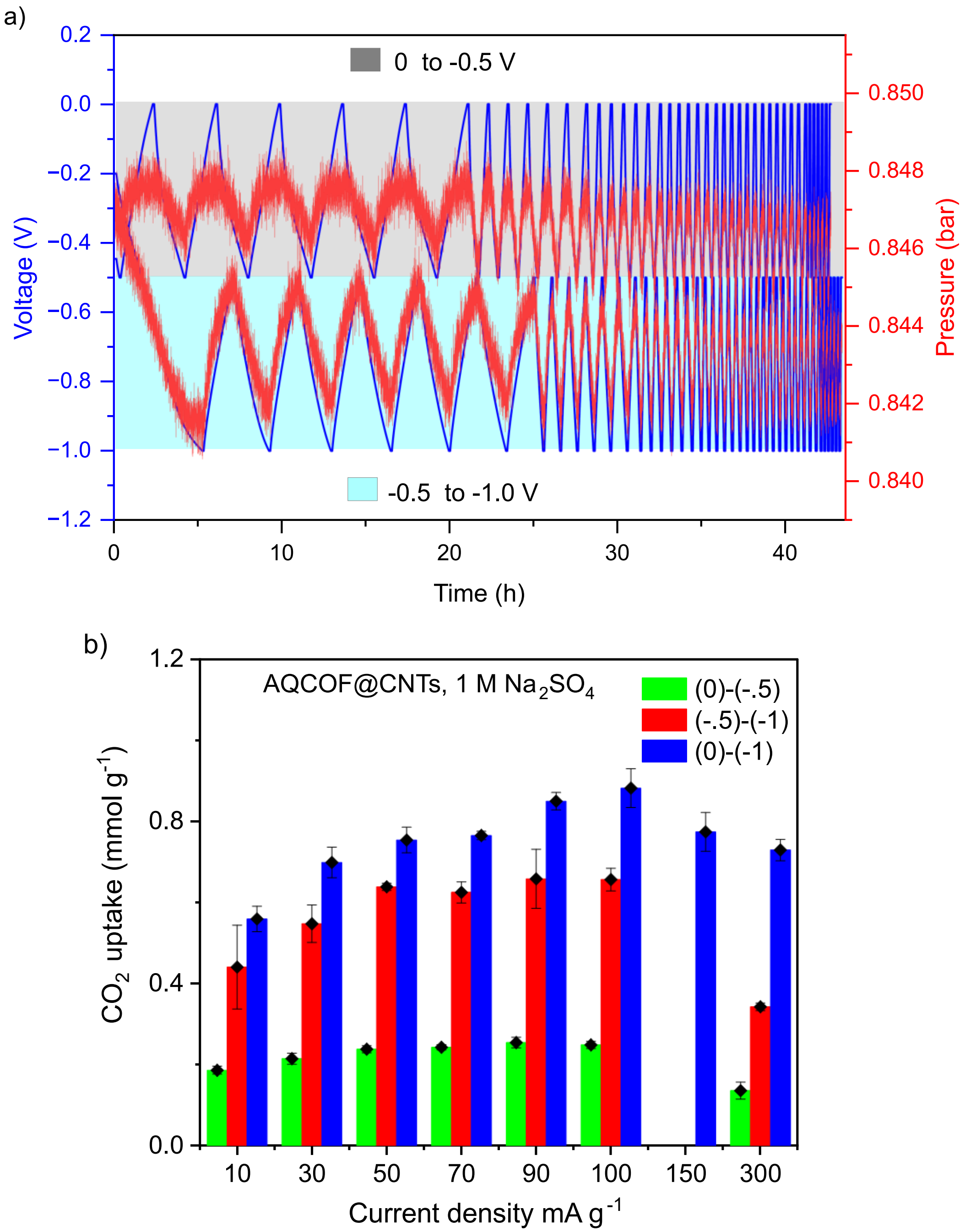

Figure S28. Effect of breaking the working voltage windows into different voltage regimes on electrochemical CO_2 capture capacity under aqueous conditions. (a) Electrochemical CO_2 uptake measurement using 'CO_2 AQCOF@CNTs / 1 M Na_2SO_4 (aq. electrolyte)/ YP80F' under 100% CO_2 atmosphere operating between the voltage window 0 to -0.5 V at different current densities from 10, 30, 50 70, 90, 100, and 300 mA g^{-1} (blue region). CO_2 uptake measurements conducted by extending voltage regime from -0.5 to -1 with all other experimental conditions unchanged (pink region). In both cases, the device was negatively charged, and a 5 min voltage hold was maintained between the charge-discharged cycles. (b) Quantified CO_2 uptake values for the respective regimes. The errors were calculated using a 95% confidence interval with the student's t-test.

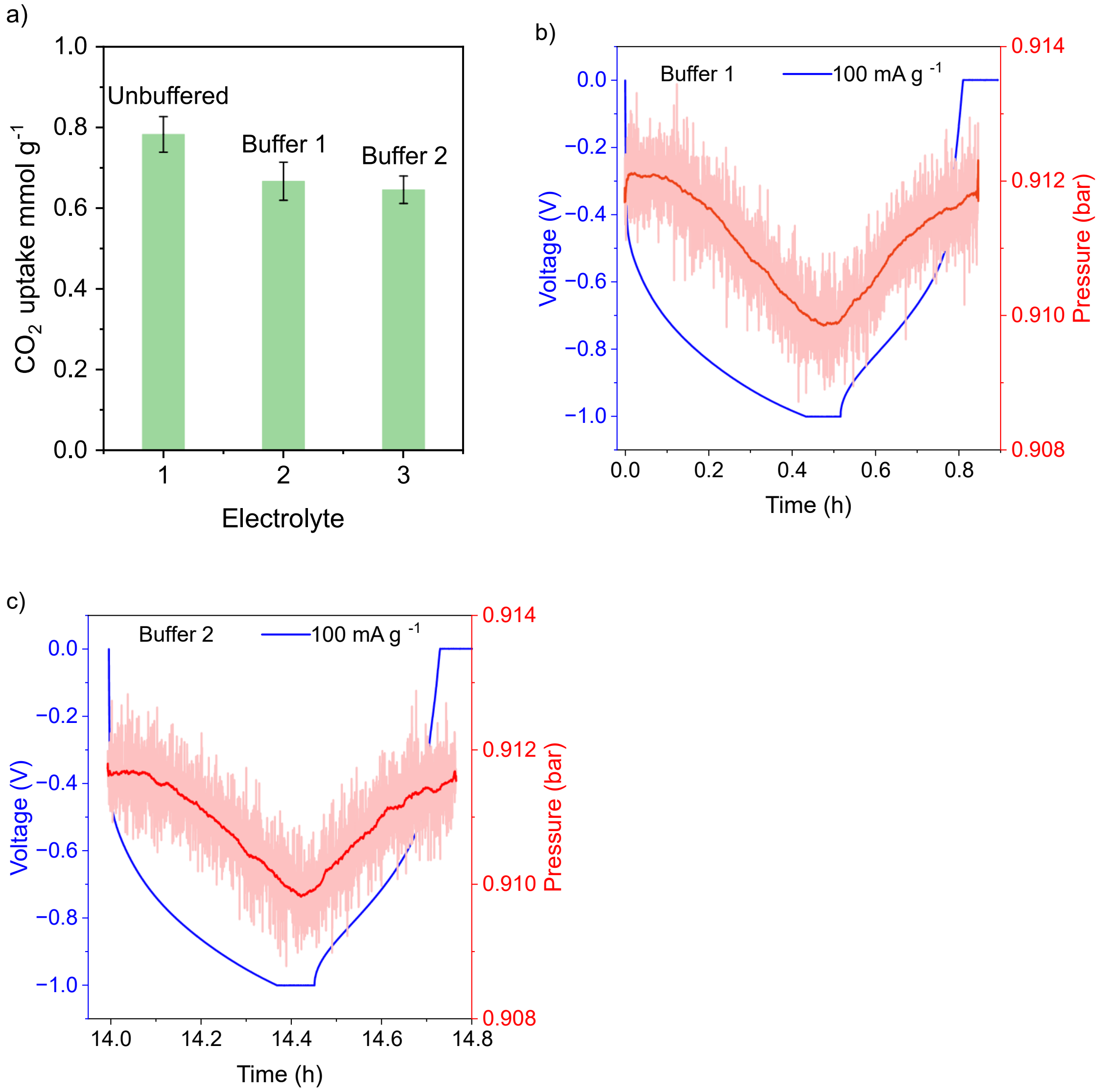

Figure 29. CO_2 uptake measurements performed in buffered electrolyte. Phosphate-buffered electrolytes (0.1 M and 0.5 M) with 0.9 M and 0.5 M Na_2SO_4, were used, maintaining a total Na^+ concentration of 2 M. (a) Comparison of CO_2 uptake capacities for unbuffered and buffered cells; (b) CO_2 pressure change cycle in 0.1 M phosphate buffer + 0.9 M Na_2SO_4 electrolyte and (c) CO_2 pressure change cycle in 0.5 phosphate buffer + 0.5 M Na_2SO_4 electrolyte.

Table S2. Comparison of CO_2 capture capacity to recently reported materials.

Cell: WE/CE configuration	Electrolyte	‡BET SSA ($m^2\ g^{-1}$)	CO_2 uptake (mmol g^{-1}) Current density mA g^{-1}					Energy used kJ/mol	Ref.
			5	10	30	100	V*		
AQCOF/YP80F	[Bmim][TFSI]	310	1.9	1.1	0.09			- -	This work
AQCOF@CNTs/YP80F	[Bmim][TFSI]	325	2.5	1.46	0.9	0.18		37	This work
†AQCOF@CNTs/YP80F	1 M Na_2SO_4	325		0.3	0.4	0.8		31	This work
^AQ@Carbon/YP80F	[Bmim][TFSI]	2000			0.4			256	[6]
BPL-AC/BPL-AC	3 M $MgBr_2$	1023					0.7		[7]
PCET	- -						0.8-1.4		[8
GP-AC/ GP-AC	1 M $NaHCO_3$	2341					0.25		[6]
YP80F/YP80F	1 M Na_2SO_4	2324			0.1			20	[3]
YP80F/Zn	1 M Na_2SO_4	2324			0.15				[4]
YP80F/Zn	1 M $ZnSO_4$	2324			0.15			51	[4]
+EMAR	- -	-						40-120	[10]
+Redox active quinones	- -	-						40-90	[11]

Anthraquinone (AQ); Coal-derived BPL carbon Calgon(BPL); Garlic root derived carbon (GR); Garlic powder derived carbon (GP); Working electrode (WE); Counter electrode (CE)

*CO_2 uptake measured at different voltages

‡ Surface area of the working electrode material

†AQCOF@CNTs contain 7 wt.% CNTs. AC is acetylene black carbon and PTFE is polytetrafluoroethylene.

+ 0.12-062 mol/mol_{amine}

+ 0.8-1.3 µmol CO_2/$\mu mol_{quinone}$

^ kJ/mol/cycle

SECTION II: Chemical Reduction and CO_2 capture

2.1.1 Synthesis of COF linker.

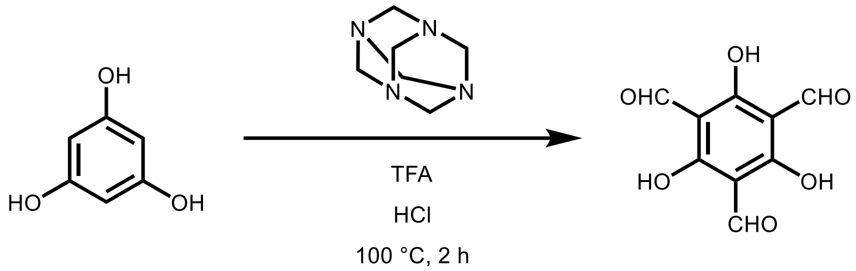

Figure S30. Synthesis of 2,4,6-triformylphloroglucinol from phloroglucinol.

Synthesis of 2,4,6-triformylphloroglucinol. This procedure was adapted from the literature.[12] In a 1 L 3-neck round-bottom flask equipped with a stir bar, hexamethylenetetraamine (24.46 g, 174.45 mmol, 2.20 equiv.) was added. The flask was placed under high vacuum and backfilled with N_2. This process was repeated a total of three times. Next, the flask was cooled to 0 ºC using an ice bath, before TFA (150 mL) was added under N_2. The mixture was stirred at 0 ºC until all solids dissolved. Then, phloroglucinol (10.00 g, 79.30 mmol, 1.00 equiv.) was added under N_2, and the ice bath was removed. The flask was then placed in a silicone oil bath and heated at 100 ºC for 2 h under N_2, at which time the solution turned dark red. After addition of 3.6 M aq. HCl (500 mL), the reaction mixture was heated at 100 ºC for 1 h, at which time the solution turned orange. At this time, the reaction mixture was filtered through celite while hot, and the filtrate was allowed to cool to room temperature, before being extracted with CH_2Cl_2 (3 × 500 mL), and the combined organic layers were dried over Na_2SO_4, filtered, and concentrated. The crude product was triturated by cold EtOH (20 mL) to afford 2,4,6-triformylphloroglucinol (2.14 g, 13% yield) as an orange-red solid. ^{1}H NMR (500 MHz, $CDCl_3$): δ 14.12 (s, 3H), 10.15 (s, 3H) ppm; ^{13}C NMR (126 MHz, $CDCl_3$): δ 192.22, 173.74, 103.03 ppm.

Mechanochemical COF synthesis

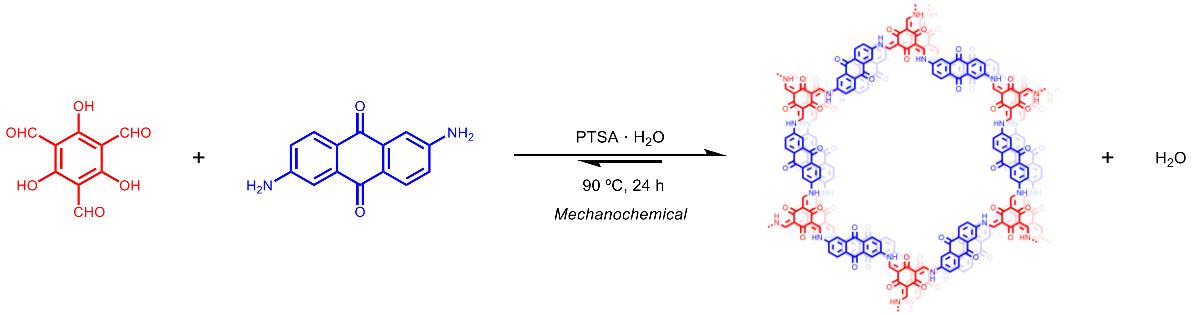

Figure S31. Synthesis of **TpDAQ-COF** from 2,4,6-triformylphloroglucinol and 2,6-diaminoanthraquinone.

Mechanochemical synthesis of TpDAQ-COF. In a 10 mL mortar, *p*-toluenesulfonic acid monohydrate (3.43 g, 18.00 mmol, 6.00 equiv.) was added and thoroughly ground using a pestle, before 2,6-diaminoanthraquinone (1.08 g, 4.50 mmol, 1.50 equiv.) was added and the mixture was thoroughly ground. Next, 2,4,6-triformylphloroglucinol (0.63 g, 3.00 mmol, 1.00 equiv.) was added and the mixture was thoroughly ground. Then, all solids were scraped to the center of the mortar and H_2O was added to the mixture one drop (~7.5 µL per drop) at a time using a micropipette. After a drop of H_2O was added, the mixture was immediately ground such that all solids began to turn into a paste-like material. This H_2O addition process was repeated until all solids turned into a homogeneous paste, which was subsequently sandwiched between two glass slides and placed in a jar with 0.4 mL H_2O at the bottom. The jar was then placed in an oven pre-heated to 90 ºC and was allowed to stand at 90 ºC for 24 h. At this time, the paste was transferred to a 250 mL Pyrex jar and was soaked in H_2O (3 × 200 mL) and *N*,*N*-dimethylacetamide (3 × 200 mL) at 60 ºC, and acetone (3 × 200 mL) at room temperature. The solid was subsequently activated by supercritical CO_2 to yield **TpDAQ-COF** as a red solid.

Chemical reduction procedure

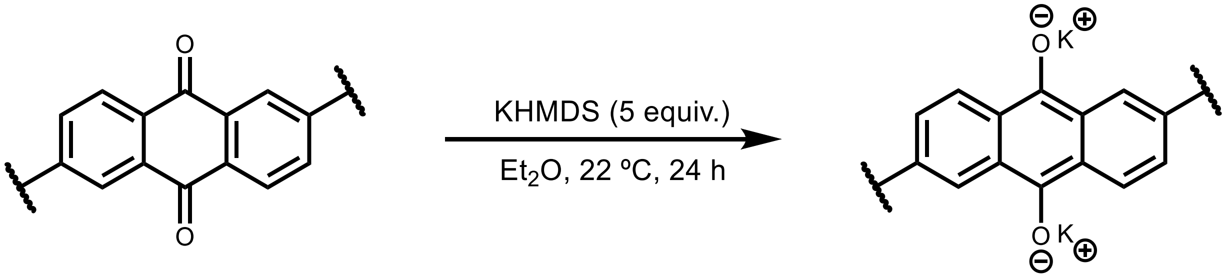

Figure S32. Chemical reduction of **TpDAQ-COF** with KHMDS.

Synthesis of reduced AQ-COF. In a N_2-filled glovebox, a solution of 10 mM KHMDS in Et_2O (10 mL, 5.00 equiv.) was added to a 20 mL vial containing the unreduced COF (20 mg). After soaking for 16 h, the solution was pipetted off and the COF was soaked in Et_2O (2 × 20 mL, 4 h each) for 8 h before being analyzed by TGA.

*This chemical reduction procedure also works for LiHMDS and NaHMDS.

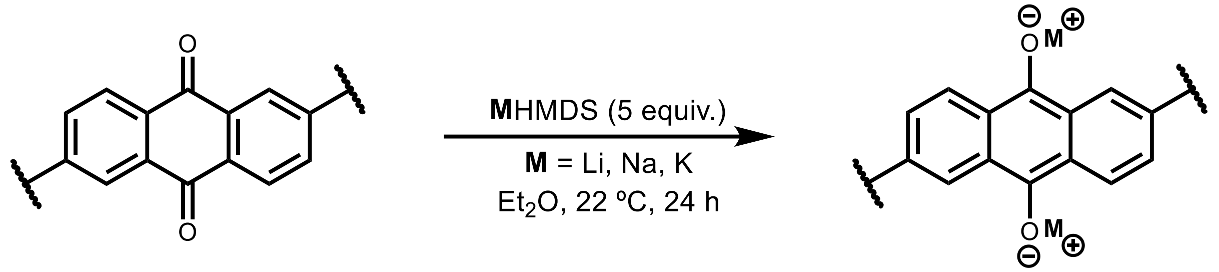

Figure S33. Chemical reduction of **TpDAQ-COF** with LiHMDS, NaHMDS, and KHMDS.

TGA procedure: In a N_2-filled glovebox, the reduced COF was wetted with benzene (~1 mL) in a 4 mL vial. Excess benzene was pipetted off, and the vial containing the wet COF was sealed and brought out of the glovebox, where it's immediately frozen in liquid N_2 and brought to the TGA room. The frozen COF was then quickly transferred to a tared pan, loaded onto the TGA, and the TGA furnace was immediately raised to put the reduced COF under a N_2 atmosphere. TGA analysis started shortly afterwards.

BET surface area analysis and CO_2 isotherm. The reduced COF was loaded into an ASAP sample tube inside a N_2-filled glovebox. The sample tube was brought out of the glovebox and BET surface area analysis and CO_2 isotherm were performed.

IR analysis. The reduced COF was loaded into an ASAP sample tube inside a N_2-filled glovebox. The sample tube was brought out of the glovebox and dosed with 1000 mbar CO_2 on the ASAP 2020 instrument for 30 min. The solids were quickly analyzed by ATR-IR.

BET surface area analysis. TpDAQ-COF was first activated by supercritical CO_2, then activated under high vacuum (< 10 μbar) at room temperature for 16 h on the ASAP 2020 instrument prior to BET surface area analysis.

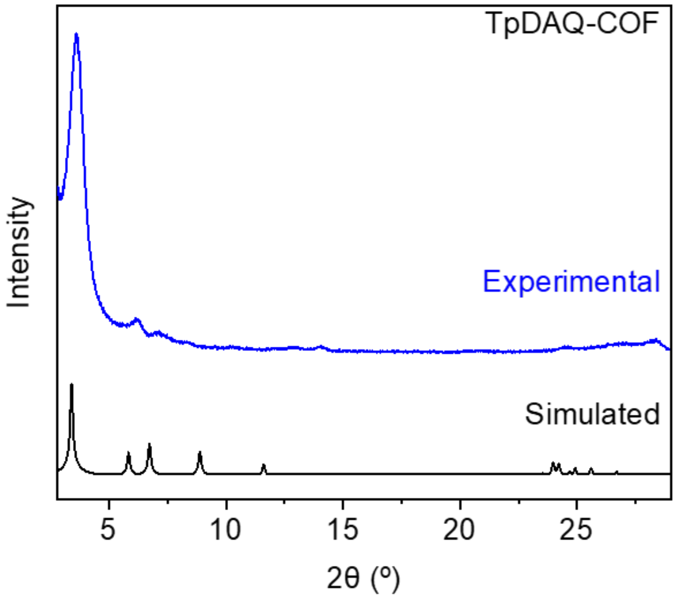

Figure S34. PXRD pattern (λ=1.5406 Å) of **TpDAQ-COF**. The simulated PXRD pattern is included for reference.[13]

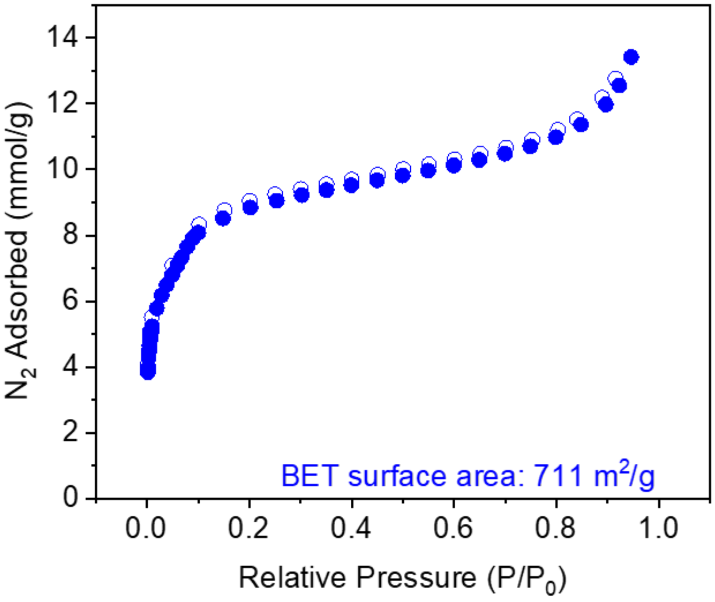

Figure S35. 77 K N_2 adsorption (filled circles) and desorption (open circles) isotherms for **TpDAQ-COF**.

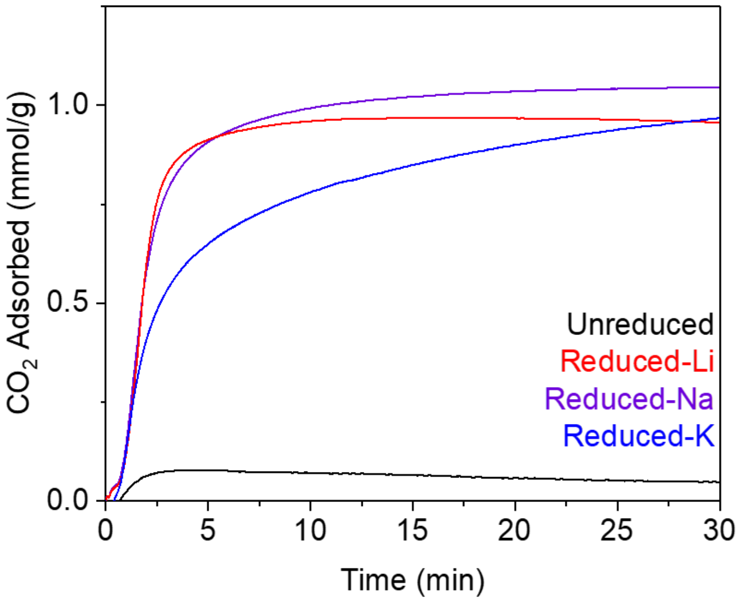

Figure S36. 5% CO_2 in N_2 adsorption isotherms at 30 ºC as measured by TGA for unreduced (black), reduced-Li (red), reduced-Na (purple), and reduced-K (blue) **TpDAQ-COF** after exposure to a flow of dry N_2 for 30 min at 30 ºC.

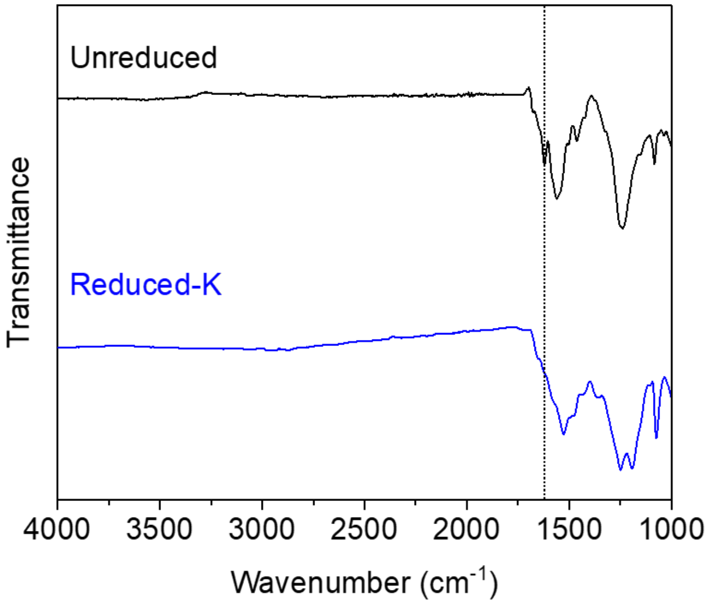

Figure S37. ATR-IR spectra for unreduced (black) and reduced (blue) **TpDAQ-COF**.

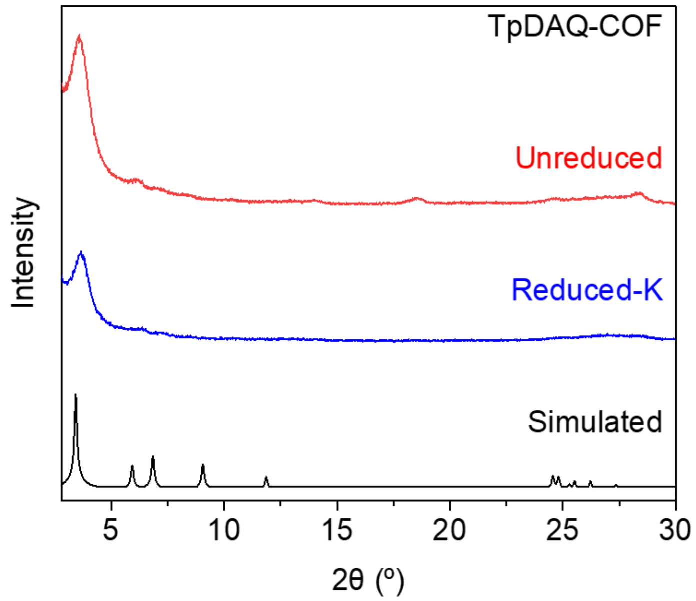

Figure S38. PXRD patterns (λ=1.5406 Å) of unreduced (red) and reduced (blue) **TpDAQ-COF**. The simulated PXRD pattern is included for reference.[2]

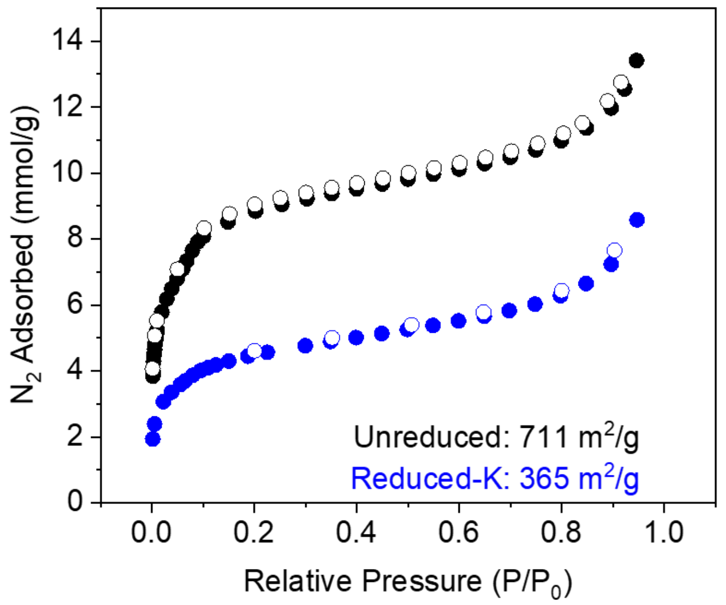

Figure S39. 77 K N_2 adsorption (filled circles) and desorption (open circles) isotherms for unreduced (black) and reduced (blue) **TpDAQ-COF**.

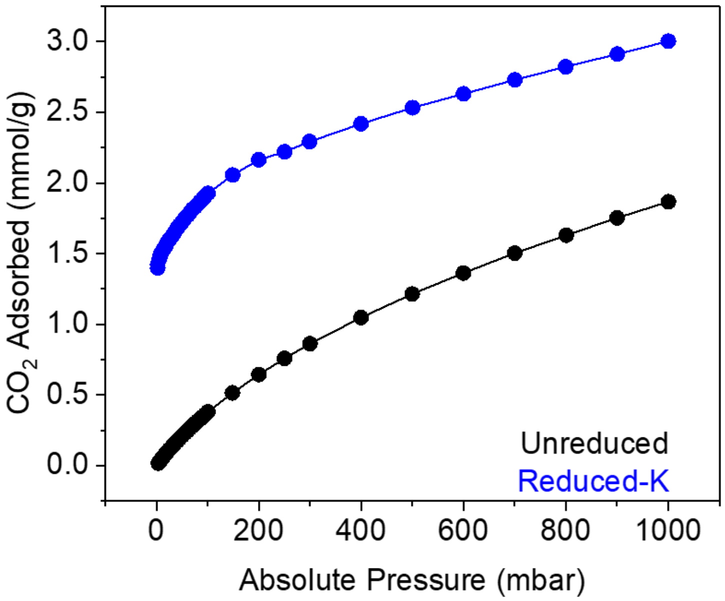

Figure S40. 30 ºC CO_2 isotherms for unreduced (black) and reduced (blue) **TpDAQ-COF**.

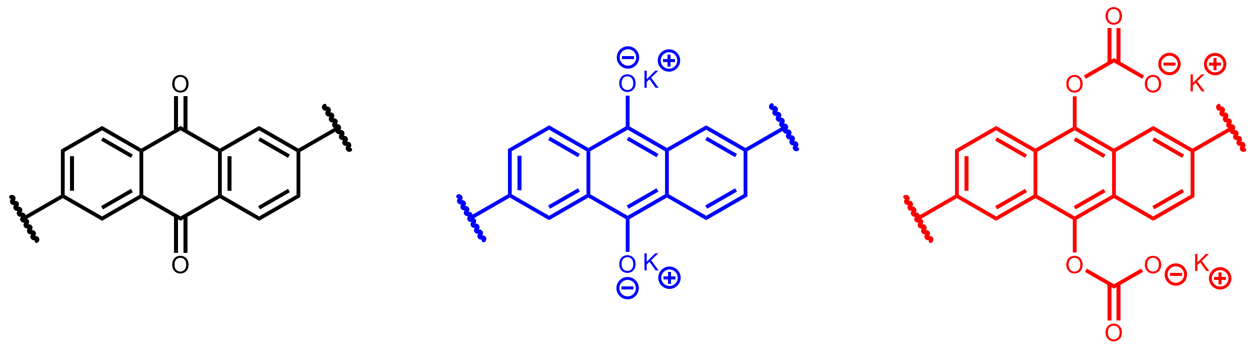

Figure S41. Chemical structures of unreduced (black), reduced (blue), and CO_2-dosed (red) **TpDAQ-COF**.

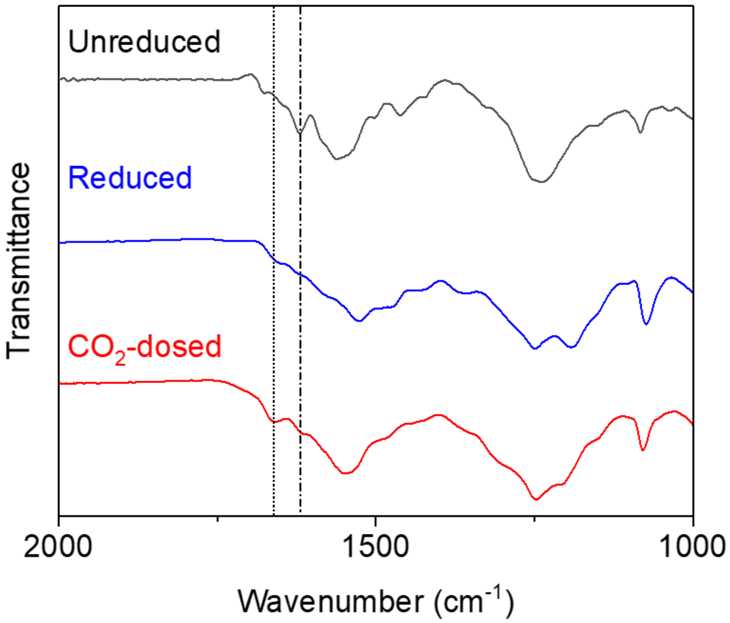

Figure S42. ATR-IR spectra for unreduced (black), reduced (blue), and CO_2-dosed (red) **TpDAQ-COF**.

References

(1) Duan, J.; Wang, W.; Zou, D.; Liu, J.; Li, N.; Weng, J.; Xu, L. P.; Guan, Y.; Zhang, Y.; Zhou, P. Construction of a Few-Layered COF@CNT Composite as an Ultrahigh Rate Cathode for Low-Cost K-Ion Batteries. *ACS Appl Mater Interfaces* **2022**, *14* (27), 31234–31244. https://doi.org/10.1021/ACSAMI.2C04831/ASSET/IMAGES/LARGE/AM2C04831_0007.JPEG.

(2) Amin, K.; Zhang, J.; Zhou, H. Y.; Lu, R.; Zhang, M.; Ashraf, N.; Yueli, C.; Mao, L.; Faul, C. F. J.; Wei, Z. Surface Controlled Pseudo-Capacitive Reactions Enabling Ultra-Fast Charging and Long-Life Organic Lithium Ion Batteries. *Sustain Energy Fuels* **2020**, *4* (8), 4179–4185. https://doi.org/10.1039/D0SE00610F.

(3) Xu, Z.; Mapstone, G.; Coady, Z.; Wang, M.; Spreng, T. L.; Liu, X.; Molino, D.; Forse, A. C. Enhancing Electrochemical Carbon Dioxide Capture with Supercapacitors. *Nature Communications 2024 15:1* **2024**, *15* (1), 1–11. https://doi.org/10.1038/s41467-024-52219-3.

(4) Xu, Z.; Liu, X.; Mapstone, G.; Coady, Z.; Seymour, C.; Wiesner, S. E.; Menkin, S.; Forse, A. C. Breaking Supercapacitor Symmetry Enhances Electrochemical Carbon Dioxide Capture. *J Am Chem Soc* **2025**, *147*, 16189–16197. https://doi.org/10.1021/JACS.5C00999/ASSET/IMAGES/LARGE/JA5C00999_0005.JPEG.

(5) Deblase, C. R.; Silberstein, K. E.; Truong, T. T.; Abruña, H. D.; Dichtel, W. R. β-Ketoenamine-Linked Covalent Organic Frameworks Capable of Pseudocapacitive Energy Storage. *J Am Chem Soc* **2013**, *135* (45), 16821–16824. https://doi.org/10.1021/JA409421D/SUPPL_FILE/JA409421D_SI_002.CIF.

(6) Hartley, N. A.; Pugh, S. M.; Xu, Z.; Leong, D. C. Y.; Jaffe, A.; Forse, A. C. Quinone-Functionalised Carbons as New Materials for Electrochemical Carbon Dioxide Capture. *J Mater Chem A Mater* **2023**, *11* (30), 16221–16232. https://doi.org/10.1039/D3TA02213G.

(7) Bilal, M.; Li, J.; Kumar, N.; Mosevitzky, B.; Wachs, I. E.; Landskron, K. Oxygen-Assisted Supercapacitive Swing Adsorption of Carbon Dioxide. *Angewandte Chemie* **2024**, *136* (39), e202404881. https://doi.org/10.1002/ANGE.202404881.

(8) Pang, S.; Jin, S.; Yang, F.; Alberts, M.; Li, L.; Xi, D.; Gordon, R. G.; Wang, P.; Aziz, M. J.; Ji, Y. A Phenazine-Based High-Capacity and High-Stability Electrochemical CO2 Capture Cell with Coupled Electricity Storage. *Nature Energy 2023 8:10* **2023**, *8* (10), 1126–1136. https://doi.org/10.1038/s41560-023-01347-z.

(9) Bilal, M.; Li, J.; Landskron, K. Enhancing Supercapacitive Swing Adsorption of CO2 with Advanced Activated Carbon Electrodes. *Adv Sustain Syst* **2023**, *7* (11), 2300250. https://doi.org/10.1002/ADSU.202300250.

(10) Wang, M.; Herzog, H. J.; Hatton, T. A. CO2 Capture Using Electrochemically Mediated Amine Regeneration. *Ind Eng Chem Res* **2020**, *59* (15), 7087–7096. https://doi.org/10.1021/ACS.IECR.9B05307.

(11) Voskian, S.; Hatton, T. A. Faradaic Electro-Swing Reactive Adsorption for CO2 Capture. *Energy Environ Sci* **2019**, *12* (12), 3530–3547. https://doi.org/10.1039/c9ee02412c.

(12) Chong, J. H.; Sauer, M.; Patrick, B. O.; MacLachlan, M. J. Highly Stable Keto-Enamine Salicylideneanilines. *Org Lett* **2003**, *5* (21), 3823–3826. https://doi.org/10.1021/OL0352714/SUPPL_FILE/OL0352714SI20030709_035619.CIF.

(13) Deblase, C. R.; Silberstein, K. E.; Truong, T. T.; Abruña, H. D.; Dichtel, W. R. β-Ketoenamine-Linked Covalent Organic Frameworks Capable of Pseudocapacitive Energy Storage. *J Am Chem Soc* **2013**, *135* (45), 16821–16824. https://doi.org/10.1021/JA409421D/SUPPL_FILE/JA409421D_SI_002.CIF.
